# Promoting flowering, lateral shoot outgrowth, leaf development, and flower abscission in tobacco plants overexpressing cotton *FLOWERING LOCUS T* (*FT*)-like gene *GhFT1*

**DOI:** 10.3389/fpls.2015.00454

**Published:** 2015-06-17

**Authors:** Chao Li, Yannan Zhang, Kun Zhang, Danli Guo, Baiming Cui, Xiyin Wang, Xianzhong Huang

**Affiliations:** ^1^Plant Genomics Laboratory, College of Life Sciences, Shihezi UniversityShihezi, China; ^2^Plant Genome Mapping Laboratory, University of Georgia, AthensGA, USA

**Keywords:** florigen, *FLOWERING LOCUS T* (*FT*), floral transition, lateral shoot, leaf morphology, abscission, tobacco

## Abstract

*FLOWERING LOCUS T* (*FT*) encodes a mobile signal protein, recognized as major component of florigen, which has a central position in regulating flowering, and also plays important roles in various physiological aspects. A mode is recently emerging for the balance of indeterminate and determinate growth, which is controlled by the ratio of *FT*-like and *TERMINAL FLOWER 1* (*TFL1*)-like gene activities, and has a strong influence on the floral transition and plant architecture. Orthologs of *GhFT1* was previously isolated and characterized from *Gossypium hirsutum*. We demonstrated that ectopic overexpression of *GhFT1* in tobacco, other than promoting flowering, promoted lateral shoot outgrowth at the base, induced more axillary bud at the axillae of rosette leaves, altered leaf morphology, increased chlorophyll content, had higher rate of photosynthesis and caused flowers abscission. Analysis of gene expression suggested that flower identity genes were significantly upregulated in transgenic plants. Further analysis of tobacco *FT* paralogs indicated that *NtFT4*, acting as flower inducer, was upregulated, whereas *NtFT2* and *NtFT3* as flower inhibitors were upregulated in transgenic plants under long-day conditions, but downregulated under short-day conditions. Our data suggests that sufficient level of transgenic cotton *FT* might disturb the balance of the endogenous tobacco *FT* paralogs of inducers and repressors and resulted in altered phenotype in transgenic tobacco, emphasizing the expanding roles of *FT* in regulating shoot architecture by advancing determine growth. Manipulating the ratio for indeterminate and determinate growth factors throughout *FT*-like and *TFL1*-like gene activity holds promise to improve plant architecture and enhance crop yield.

## Introduction

Plants sense multiple environmental cues and endogenous signals to determine the appropriate timing of flowering, which is an orchestrated process through the integration of multiple environmental cues and endogenous signals. Genetic and molecular analyses of flowering-time mutants in *Arabidopsis* have established the current model, in which five major pathways mainly control the transition from the vegetative to reproductive phase. The photoperiodic and vernalization pathways are responsive to the appropriate environmental conditions, whereas the autonomous, gibberellin, and age pathways reflect the internal status of plants (Srikanth and Schmid, 2011; Yamaguchi and Abe, 2012), which all converge on the ‘hubs’ known as the integrator genes. Among them, *FLOWERING LOCUS T* (*FT*) and its paralog *TWIN SISTER OF FT* (*TSF*), encodes a ∼20 kDa globular proteins of the phosphatidylethanolamine-binding protein (PEBP) family, which has a central position in mediating the onset of flowering (Kardailsky et al., 1999; Kobayashi et al., 1999; Yamaguchi and Abe, 2012; Hiraoka et al., 2013). *FT* as well as TSF proteins including tomato SINGLE FLOWER TRUSS (SFT) and rice HEADING DATE 3a (Hd3a; Lifschitz et al., 2006; Corbesier et al., 2007; Mathieu et al., 2007; Tamaki et al., 2007; Notaguchi et al., 2008), nicknamed florigen, were produced in the phloem companion cells. They are subsequently transported to the shoot apical meristem (SAM), where they form a complex involving a bZIP transcription factor FLOWERING LOCUS D (FD) to activate the expression of floral meristem identity genes, including *SUPPRESSOR OF OVEREXPRESSION OF CONSTANS1* (*SOC1*), *APETALA1* (*AP1*), and *LEAFY* (*LFY*; Abe et al., 2005; Wigge et al., 2005; Yoo et al., 2005; Kaufmann et al., 2010), which are important regulatory of hubs in the control of flowering time.

Phylogenetic studies of PEBP-like genes in angiosperms revealed that they fall into three subfamilies: the *FT*-like, the *TEMINAL FLOWER1* (*TFL1*)-like and the *MOTHER OF FT AND TFL1* (*MFT*)-like (Chardon and Damerval, 2005; Hedman et al., 2009). *FT*-like and *TFL1*-like genes modulate flowering transition and inflorescence architecture (Kobayashi et al., 1999; Hanzawa et al., 2005; Ahn et al., 2006), but their functions in flowering control are opposite. *FT* promotes the transition to reproductive development and flowering, whereas TFL1 represses this transition.

Numerous studies have concluded, *FT* orthologs possessing floral inductive function in woody perennials (Hisada et al., 1997; Endo et al., 2005; Böhlenius et al., 2006; Hsu et al., 2006; Carmona et al., 2007; Kotoda et al., 2010; Song et al., 2013); grasses (Yan et al., 2006; Tamaki et al., 2007; Kikuchi et al., 2009; Meng et al., 2011; Wu et al., 2013; Coelho et al., 2014); legumes (Kong et al., 2010; Ono et al., 2010; Hecht et al., 2011; Laurie et al., 2011); ornamental (Hayama et al., 2007; Hou and Yang, 2009; Imamura et al., 2011), CsFTL3 from chrysanthemum (*Chrysanthemum seticuspe*; Oda et al., 2012; Xiang et al., 2012; Li et al., 2013); and others such as BvFT2 from sugar beet (*Beta vulgaris*; Pin et al., 2010), NtFT4 from tobacco (*Nicotiana tabacum*; Harig et al., 2012), StSP3D form potato (*Solanum tuberosum*; Navarro et al., 2011), AcFT2 from onion (*Allium cepa*; Lee et al., 2013), PaFT from avocado (*Persa americana*; Ziv et al., 2014), LsFT from lettuce (*Lactuca sativa*; Fukuda et al., 2011; Above information was listed in Supplementary Table S1). Previously study suggests a conserved ancestral function of *FT*-like proteins in transmitting inductive signals in plants. However, recent studies showed that *FT*-like genes in numerous species play important roles in various physiological aspects other than flowering (Pin and Nilsson, 2012). In *Arabidopsis*, *FT* and *TSF* regulates stomatal guard cells opening by activating H^+^-ATPase (Kinoshita et al., 2011), meristem maintenance in cooperation with *SHOOT MERISTEMLESS* (*STM*) and FD during inflorescence development (Smith et al., 2011), and prevention of indeterminate growth, floral reversion and aerial rosette (Melzer et al., 2008). *FT* and *TSF* modulate lateral shoot outgrowth in *Arabidopsis*, and link the floral transition and lateral shoot development to maximize the reproductive success of a plant (Hiraoka et al., 2013). *FT* has also been demonstrated to be involved in multiple steps of axillary bud development, likely to coordinate axillary shoot development with flowering (Niwa et al., 2013). Ectopic overexpression of *FT* in cotton through virus-induced flowering uncouples flowering from photoperiodic regulation and promotes determinate growth habit in all aerial organs (McGarry and Ayre, 2012). In tomato, *SINGLE FLOWER TRUSS* (*SFT*) regulates reiterative growth and termination of shoots, influences leaf maturation, compound leaf architecture, stem growth, and abscission zone formation (Shalit et al., 2009). Florigen is thus established as a plant protein functioning as a general growth hormone. Also, allelic variation at the *SFT* locus is implicated in heterosis of yield (Krieger et al., 2010), suggesting a single overdominant gene may improve productivity in other agricultural organisms, which supports the overdominance model for heterosis. *PtFT1* controls short-day (SD) induced growth cessation and bud set in autumn (Böhlenius et al., 2006). Some members of *FT*-like gene family modulate growth of underground storage organs. *StSP6A* functions as a mobile ‘tuberigen’ that induces the photoperiod-sensitive process of tuberization in potato (Navarro et al., 2011), and *AcFT1* and *AcFT4* play role in bulb formation in onion (Lee et al., 2013).

The *Gossypium* (Cotton) is one of the most important cash crops worldwide, having a large impact on our economy and everyday life. *Gossypium* species are naturally a photoperiodic that does not flower until the shorter days of late summer or fall. Domestication of the two allotetraploid that comprise the majority of world-wide cultivations, *Gossypium hirsutum* and *G. barbadens* gradually lose their photoperiod sensitivity (McGarry and Ayre, 2012). Cotton originated from a tropical region, and its growth is very sensitive to low temperature and soil conditions in temperate cultivation regions. Flowering earliness is an important objective in most cotton breeding programs. However, the molecular mechanisms regulating the transition from vegetative to reproductive growth in cotton are less well characterized than in other plant species, mostly due to the complexity of cotton genome and scarcity of cotton flowering time mutants. In previous study, we isolated and characterized an *FT*-like gene *GhFT1* from *G. hirsutum*, and we investigated its temporal and spatial expression profile during cotton multiple develop stages (Guo et al., 2015). Overexpression of *GhFT1* in *Arabidopsis* obviously generated early flowering phenotypes in both LD and SD conditions, showing that *GhFT1* is a putative *FT* ortholog in *G. hirsutum* that regulates floral transition, similar to *Arabidopsis* (Guo et al., 2015). In this study, we further dissected its roles by ectopic expression of *GhFT1* in wild-type (WT) tobacco. As expected, *GhFT1* obviously promotes the floral transition in transgenic tobacco plants by producing terminal flower. However, boosting flowering is just one of the pleiotropic functions of *GhFT1*. In addition to precocious flowering, we observed that tobacco plants carrying *35S::GhFT1* had more lateral shoots outgrowth at the base, axillary buds at rosette axil, altering leaves morphology and causing flower abscission. Our data suggests that sufficient level of transgenic cotton *FT* homolog might disturb the balance of endogenous *FT*-like proteins and disorder the ratio of inducer and repressors, resulting in inflorescence and plant architecture change.

## Materials and Methods

### Plant Materials and Growth Conditions

The seeds of *N. tabacum* cv. NC89 and *N. benthamiana* preserved in our lab were surface-sterilized for 20 min with 2.8% sodium hypochlorite solution containing 0.1% surfactant (Triton X-100, Sigma-Aldrich, Munich, Germany), and rinsed several times with sterile water. Then seeds were stratified for 3 days at 4°C in darkness and then plated on the Petri dishes with half-strength Murashige and Skoog (MS) medium containing MS salt (pH 5.7; Duchefa, Haarlem, the Netherlands) mixture, 1% (w/v) sucrose and 0.8% (w/v) agar. Petri dishes were then placed in light growth incubator at 28°C for 15 days under SD conditions (8 h light/16 h dark). The aseptic seedlings of *N. tabacum* for transformation were then transferred into a sterile flask containing half-strength MS medium at 28°C for another 30 days. The *N. benthamiana* seedlings for transient expression assay were transplanted into soil after germination and grown in phytotron under long-day (LD) conditions (16 h light/8 h dark), and the light intensity for tobacco growth is 200 μmol m^-2^ s^-1^.

### Constructions of Overexpression Vectors

*35S::GhFT1*, *35S::GFP*, and *35S::GhFT1-GFP* constructs were the same vectors that were used in Guo’s study (Guo et al., 2015). We first replaced the *GUS* fragment in the binary vector pCAMBIA1301 (CAMBIA, Canberra, ACT, Australia) by 525 bp of *GhFT1* encoding sequence (digested with *Nco* I and *Bst* EII restriction, respectively) to construct *pCAMBIA1301-GhFT1*. The 5.7 kb upstream sequence of *Arabidopsis thaliana FT* was amplified by polymerase chain reaction (PCR) using pDONR207-*8.1kbAtFTpro* plasmid as template and next cloned into the *Pst* I and *Nco* I restriction site of the *pCAMBIA1301-GhFT1* vector to construct the *5.7kbAtFTpro::GhFT1* plasmid. *35S::GhFT1*, *35S::GhFT1-GFP* and *5.7kbAtFTpro::GhFT1* were all transfected into *Agrobacterium tumefaciens* GV3101(pMP90RK) by electroporation.

### Tobacco Transformation

Tobacco plants (*N. tabacum* cv. NC89) were transformed with *35S::GhFT1*, *35S::GhFT1-GFP*, and *5.7kbAtFTpro::GhFT1*, respectively, using *Agarobacterium*-mediated tobacco transform of leaf disks method (Horsch et al., 1986). We generated numerous homozygous transgenic lines carrying *35S::GhFT1*, *35S::GhFT1-GFP*, and *5.7kbAtFTpro::GhFT1*. For phenotypic and gene expression analysis, all transgenic lines and WT tobacco plants were sown in pot containing soil and cultivated in phytotron under LD and SD conditions with 200 μmol m^-2^ s^-1^ light intensity, respectively.

### Subcellular Location Analysis

For analysis of the subcellular localization of GhFT1 protein using GFP reporter gene, the *35S::GhFT1-GFP* construction (Guo et al., 2015) was transformed into *N. tabacum* cv. NC89 plants stably by *A. tumefaciens* strain GV3101(pMP90RK). Hypocotyl of selected *35S::GhFT1-GFP* homozygous transformants were used to detect GFP fluorescent by confocal lase scanning microscopy (CLSM; Zeiss, LSM510, Jena, Germany).

The transient expression assays in tobacco were performed according to the method described by Voinnet et al. (2003). The *A. tumefaciens* strain GV3101 (pMP90RK) carrying *35S::GhFT1-GFP* was grown at 28°C in LB medium with kanamycin and rifampicin to OD_600_ = 0.5-0.6. The agrobacteria cells were centrifuged and re-suspended in 10 mmol L^-1^ MgCl_2_, 10 mmol L^-1^ MES-KOH (pH 5.7) and 150 μmol L^-1^ acetosyringone to OD_600_ = 0.5. The agrobacteria cells were left to standing for 3 h at room temperature and then infiltrated into the abaxial side of leaves of 4-weeks-old *N. benthamiana* plants. After 3–5 days the infiltrated leaves were selected to detect GFP fluorescent by CLSM.

### Gene Expression Analysis

Total RNA was isolated using Trizol reagent (Invitrogen, Carlsbad, CA, USA) according to the manufacture’s protocol. The cDNA synthesis reactions were performed using the Superscript^®^ First-Strand Synthesis System (Invitrogen, Carlsbad, CA, USA) according to the manufacturer’s instructions with 1 μg of total RNA per reaction used as template. qRT-PCR was carried out using Applied Biosystems 7500 Fast Real-Time PCR System and Fast SYBR^®^ Green Master Mix (Life Technologies, Foster City, CA, USA) to detect the expression of *GhFT1* and endogenous genes in transgenic tobacco lines and WT plants. Primers information used in this research are listed in Supplementary Table S5. NtActin-F and NtActin-R were used to amplify the *NtActin* gene (GenBank accession no. U60495), which was used as an internal control. At least three replicate assays were performed with independently isolated RNA for all experiments. Each RT reaction was loaded in triplicate for qRT-PCR analysis. qRT-PCR data were analyzed using the PCR analysis program 7500 software v2.0.6 (Life Technologies, Foster City, CA, USA).

Semi-quantitative RT-PCR was performed as described by Xu et al. (2013). Gene-specific primers GhFT1-F2 and GhFT1-R2 were used to analyze the expression of *GhFT1* in *35S::GhFT1-GFP* transgenic tobaccos, and *NtActin* was used as an internal control. Amplification was performed for 28 cycles at 94°C for 30 s, 58°C for 30 s, and 72°C for 30 s. PCR products were subsequently separated on a 1.2% (w/v) agarose gel, then stained with ethidium bromide and photographed under UV light.

### Chlorophyll Content Determination

Determination of chlorophyll content in transgenic plants grown under LD and SD conditions were estimated according to the method described by Xu et al. (2013). 0.1 g plant tissue was homogenized in 80% acetone and incubated in dark for 6 h. The homogenate was centrifuged at 10,000 rpm for 10 min. Supernatant obtained was read at 649, 665 nm in Spectra Max plus-384 (Molecular device, USA).

### Leaf Mass Per Area (LMA) Measurements

Leaf area was measured using paper-cutting method described by Hattersley (1984) with some modification. We digitally photographed leaves of different transgenic tobacco lines and WT plants. Photos of different lines were printed randomly with A4 paper (No. 3954, Deli, Ningbo, China). Then the printed photos were cut off carefully. Weight of leaves (W1) and their corresponding cut-off papers (W2) were weighed. Then LMA was calculated using the following formula: LMA = (7 × W1)/(1000 × W2) cm^-2^. At least three replicate assays were performed independently in this experiment.

### Photosynthetic Rate Curve

The photosynthetic rate of tobacco plants under LD and SD conditions were measured by LI-6400 (LI-COR Inc., Lincolin, NE, USA) with auto-measure program. The CO_2_ concentrations in sample phytotron were controlled at 400 μmol CO_2_ mol^-1^. And different red-blue light intensity 2000, 1800, 1500, 1200, 1000, 800, 500, 300, 200, 100, 50, and 0 μmol m^-2^ s^-1^ were applied to measure the net CO_2_ uptake rate. Light curve data were analyzed using the built-in program in LI-6400 system.

## Results

### Cytoplasm and Nucleus Location of GhFT1 Protein

**FIGURE 1:**
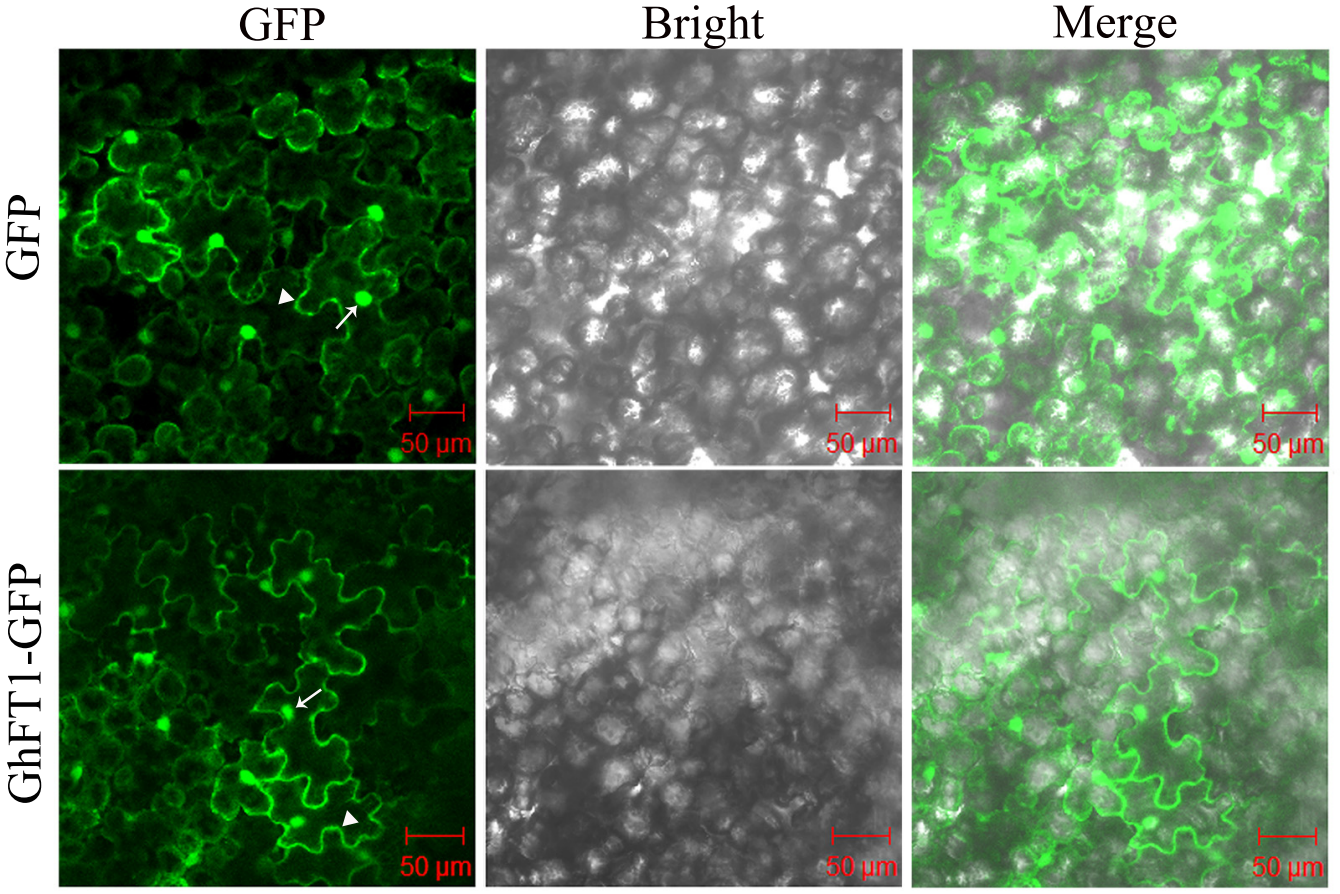
**Nucleus and cytoplasm subcelluar location of GhFT1-green fluorescent protein (GFP) in *N. benthamiana*.** Micrographs showing cells expressing *GFP* (control, upper lane) or GhFT1-GFP (bottom lane) fusion protein, which were examined under fluorescent-field illumination (left) to examine GFP fluorescence, and under bright-field illumination (middle), and by confocal microscopy (right) for an overlay of bright and fluorescent illumination. Arrow, plasma membrane; triangle, nucleus.

We previously confirmed that GhFT1 located in the cytoplasm and nucleus by detecting the fused green fluorescence protein (GFP) in the *Arabidopsis* root cells carrying *35S::GhFT1-GFP* (Guo et al., 2015). In this study, we initially generated up to 12 independent *35S::GhFT1-GFP* transgenic tobacco plants by transformation with *A. tumefaciens*. We next to observed the green fluorescence using CLSM. As expected, root tip cells expressing GhFT1-GFP fusion protein revealed strong fluorescence in the nucleus, and GFP signal was also obvious in the cell membrane (Supplementary Figure S2), which was similar to that of the *35S::GhFT1-GFP* transgenic *Arabidopsis* seedling (Guo et al., 2015). To exclude the possibility of cell wall association of GhFT1-GFP, we performed the plasmolysis assay by sucrose treatment. However, we were not able to observe an ideal picture of plasmolysis due to the very thick cell wall of tobacco root tips. Furthermore, the resulting construct of *35S::GhFT1-GFP* (Supplementary Figure S1A) was transformed transiently into *N. benthamiana*. We next monitored the subcellular location of the GhFT1-GFP fusion protein by CLSM in the leaf epidermal cells of *N. benthamiana*. Green fluorescence was detected in the peripheral cytoplasm (surrounding the vacuole) as well as in the nucleus, which was similar to the cells expressing GFP alone (**Figure 1**). As our previous report (Guo et al., 2015), we further confirmed that GhFT1 localized in both the cytoplasm and nucleus in plant cells.

We next transferred all the *35S:GhFT1-GFP* transgenic tobacco plants into pots containing soil. Under SD conditions, these transgenic plants flowered at 63 ± 6.6 days after sowing with 14.5 ± 0.6 leaves (Supplementary Figure S3A), compared with 94.5 ± 3.3 days in the WT tobacco plants with 15.7 ± 0.5 leaves (Supplementary Table S2). To investigate whether *GhFT1* was highly expressed in the *35S::GhFT1-GFP* transgenic lines, semiquantitative reverse transcription-polymerase chain reaction (RT-PCR) was performed. As shown in Supplementary Figure S3B, *GhFT1* was expressed in all the selected transgenic lines, and the flowering time was positively correlated with expression level of *GhFT1* in transgenic lines.

### Ectopic Expression of *GhFT1* Promoted Flowering in *N. tabacum*

**FIGURE 2:**
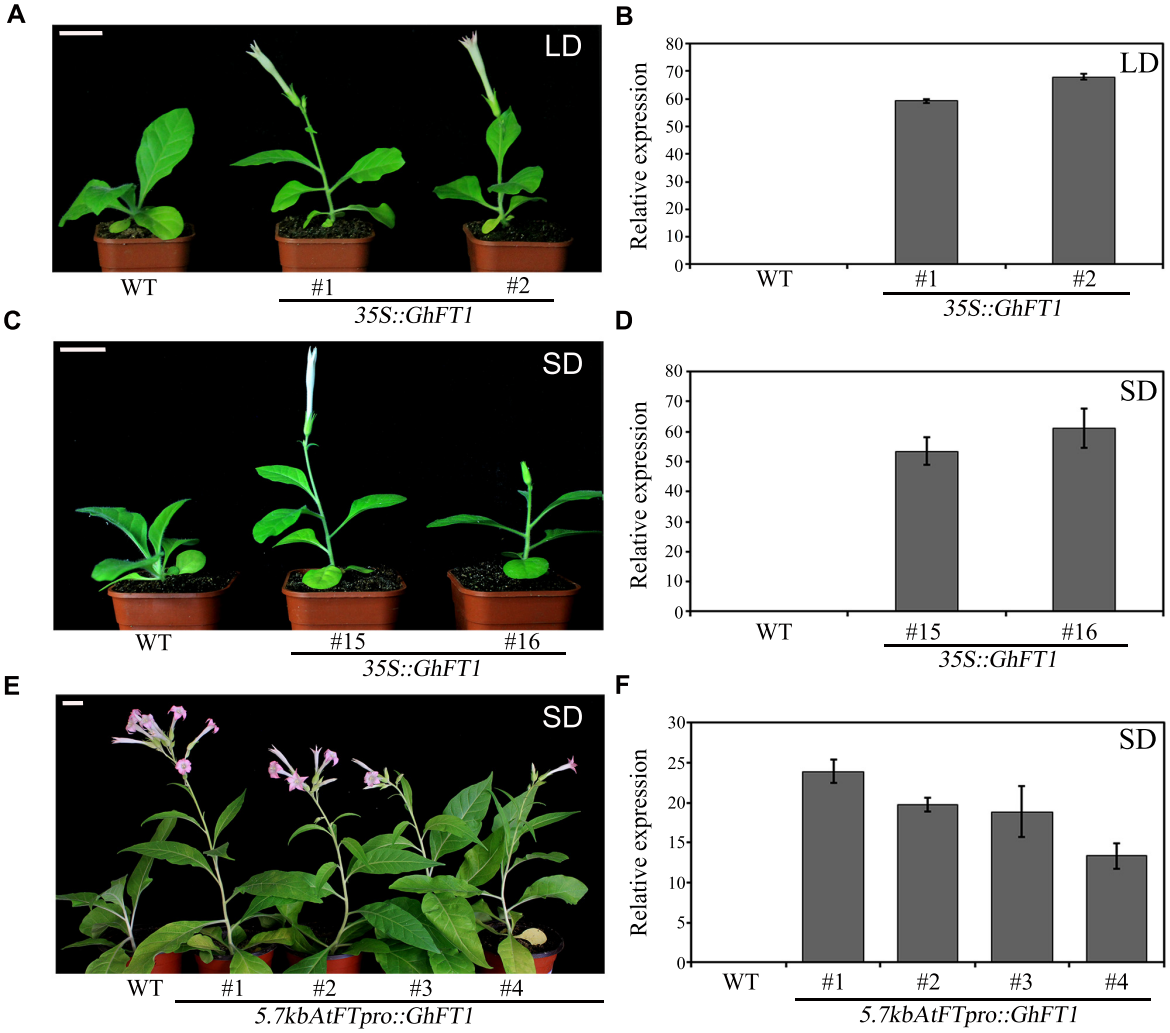
**Phenotype analysis of transgenic tobacco (*Nicotiana tabacum*) lines that ectopically expressed *GhFT1*. (A)** Appearance of 41 days wild-type (WT) tobacco and *35S::GhFT1* transgenic tobacco line 1 and line 2 grown in phytotron under long-day (LD; 16 h light/8 h dark) conditions. **(B)** Detection of *GhFT1* expression by quantitative real-time PCR (qRT-PCR) in the *35S::GhFT1* transgenic lines and WT control under LD conditions. **(C)** Appearance of 54 days WT tobacco and *35S::GhFT1* transgenic tobaccos line 15 and line 16 grown in phytotron under short-day (SD; 8 h light/16 h dark) conditions. **(D)** Detection of *GhFT1* expression by qRT-PCR in the *35S::GhFT1* transgenic lines and WT control under SD conditions. **(E)** Appearance of 53 days WT tobacco and *5.7kbAtFTpro::GhFT1* transgenic tobacco lines grown in phytotron under SD conditions. **(F)** Detection of *GhFT1* expression by qRT-PCR in the *35S::GhFT1* transgenic lines and WT control under LD conditions. Scale bars: 3.0 cm. Values are mean ± SE of results from three independent replicates (*n* = 3).

Our previous research showed that overexpression of cotton *GhFT1* in *Arabidopsis* caused early flowering both LD and SD conditions (Guo et al., 2015). To explore the potential of *GhFT1* in the regulation of flowering in tobacco, this gene was overexpressed in *N. tabacum* under the control of the strong and constitutive cauliflower mosaic virus (CaMV) 35S promoter by transformation with *35S::GhFT1* construct (Supplementary Figure S1B). We obtained numerous transgenic lines from two times of independent transform assays, and all of them were confirmed by PCR (data was not provided). The majority of *35S::GhFT1* primary transformants flowered much early than the WT, both in terms of time and the number of leaves before flowering (Supplementary Table S2). In the homozygous T3 plants, 14 showed significantly early flowering phenotype under LD (line 1 and line 2 are shown as an example in **Figure 2A**), and 12 also showed precocious flowering compared with the WT plants under SD conditions (line 15 and line 16 are shown as an example in **Figure 2B**). In LD conditions, the flowering time in the *35S::GhFT1* transgenic lines was 45.8 ± 4.8 days after sowing by producing 8 ± 0.7 leaves, compared with 106.3 ± 4.8 days by producing 16 ± 0.8 leaves in the WT (Supplementary Table S2). Likewise, under SD conditions, the flowering time of the *35S::GhFT1* transgenic lines was about 57.2 ± 4.9 days by producing 7 ± 0.9 leaves, compared with 94.5 ± 3.3 days by producing 15.7 ± 0.5 leaves in the WT siblings (Supplementary Table S2). In addition, transgenic lines had rapidly elongated internodes and reduced internodal length, and thereby developed dwarf stature when flowering than the controls under both conditions.

Previously, it has been shown that a transgene consisting of 5.7-kb sequence upstream of the *Arabidopsis FT* translation start site fused to the *FT* cDNA was sufficient to rescue the late flowering phenotype of *ft-10* plants grown under inductive extended SD conditions (Adrian et al., 2010). To further analyze the function of *GhFT1* in promoting flowers, we designed the *5.7kbAtFTpro::GhFT1* construction by using 5.7-kb *Arabidopsis FT* gene promoter fused to the *GhFT1* cDNA (Supplementary Figure S1C). We next generated up to 14 independent transgenic lines expressing *GhFT1* cDNA by transformation with *A*. *tumefaciens*. All of them flowered earlier than the WT plants under non-inductive SD conditions (**Figure 2E**). The average flowering time for these *5.7kbFTpro::GhFT1* transgenic lines was approximately 50.5 ± 2.1 days average by producing 13.5 ± 1.0 leaves, whereas the flowering time in WT sibling was 94.5 ± 3.3 days by producing 15.7 ± 0.5 leaves (Supplementary Table S2). These data combined with previous report by Guo et al. (2015) further supported that the gene product of *GhFT1* function as a floral activator to promote flowering in cotton.

To explore whether the early flowering phenotype correlated with *GhFT1* expression in the transgenic tobacco lines, we used quantitative Real-time PCR (qRT-PCR) methods to analyze gene expression level. As shown in **Figures 2B,D,F**, higher *GhFT1* expression was observed in early flowering transgenic lines more than in those with a less phenotype, whereas no *GhFT1* expression was seen in WT.

### Ectopic Expression of *GhFT1* Caused Lateral Shoot Outgrowth in Tobacco

**FIGURE 3:**
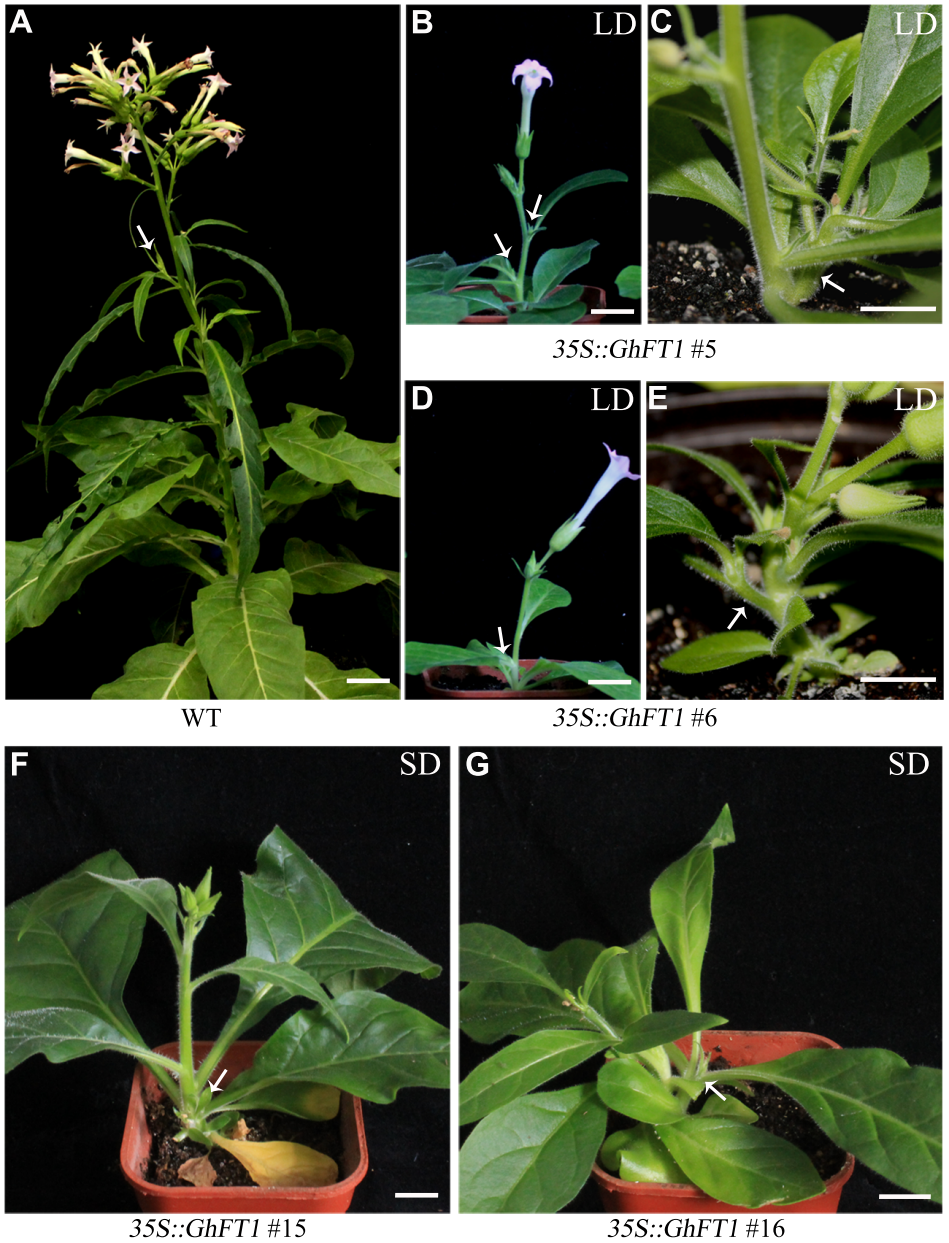
**Ectopical overexpression of *GhFT1* promotes lateral shoot outgrowth in tobacco. (A)** The non-transformed WT tobacco plant develops lateral shoots (white arrow) at the axillary buds after flowering. **(B,D)** The *35S::GhFT1* transgenic tobacco lines developed more axillary buds (white arrows) after flowering under LD conditions. **(C,E)** The *35S::GhFT1* transgenic tobacco plants developed lateral shoots (white arrows) at stem base under LD conditions. **(F,G)** The *35S::GhFT1* transgenic tobacco plant lines developed lateral shoots (white arrows) from stem base at early buds stage under SD conditions. Scale bars: 2 cm.

*Nicotiana tabacum* is an annually grown herbaceous plant with little branches. Many flowered inflorescences arise at the terminal after floral transition of the plant (Amaya et al., 1999). Axillary buds are formed at the axillae of foliage leaves, and will further develop into an inflorescence shoot (**Figure 3A**). Surprisingly, we also observed that the *35S::GhFT1* transgenic plants produced more axillary buds after floral transition under LD conditions, but these axillary buds could not further develop into lateral shoots (**Figures 3B,D**), whereas the WT tobacco plants could. Conversely, more lateral shoots were generated at the base of transgenic plants, which do not usually appear in the WT plants of laboratory accession, *N. tabacum* cv. NC89 (**Figures 3C,E**; Supplementary Figures S4A,B). Under SD conditions, the formation of axillary buds in all the *35S::GhFT1* transgenic plants were not as obvious as in the LD conditions. They also could not develop into lateral shoots finally, and more lateral shoots were generated from stem base at early buds stage (**Figures 3F,G**; Supplementary Figure S4C). These observations suggested that ectopic expression of *GhFT1* in tobacco could modulate lateral shoot outgrowth and axillary bud set in additional to floral transition.

### Overexpression of *GhFT1* Influenced Leaf Morphology in Tobacco

**FIGURE 4:**
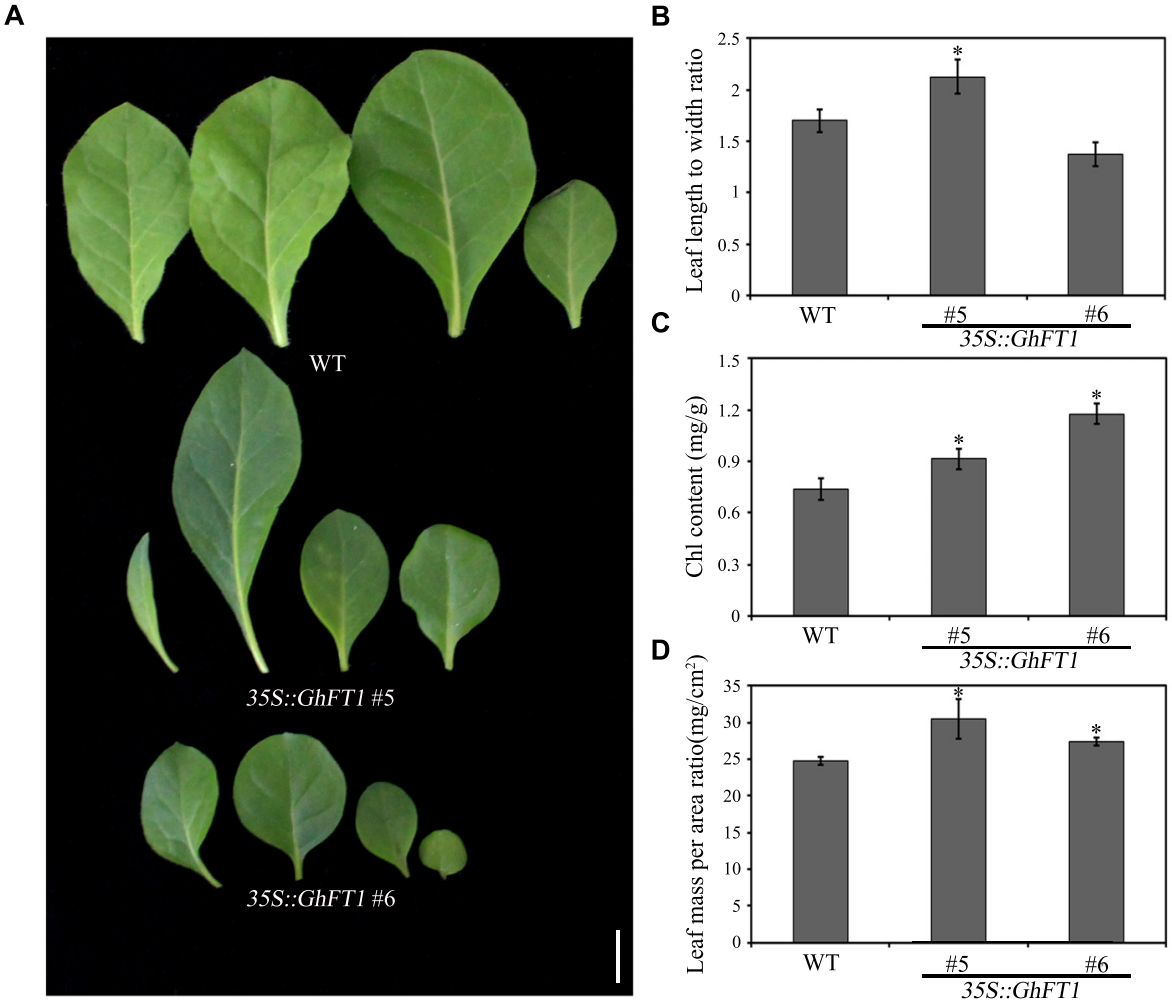
**Overexpression of *GhFT1* in tobacco has a powerful influence on leaf development. (A)** Comparison of apical (left), medial (middle two), and basal (bottom) leaves among WT plant and the *35S::GhFT1* transgenic line 5 and line 6 (at 7 weeks) under LD conditions. Scale bar: 1 cm. The ratio of leaf length to width (L/W; **B)**, chlorophyll content **(C)** and Leaf mass per area (LMA; **D)** were determined among WT plant and the *35S::GhFT1* transgenic tobacco line 5 and line 6 (at 7 weeks) under LD conditions, respectively. Data represent the mean ± SE of three independent experiments (*n* = 4). The asterisks indicate significant differences compared with the WT plants (*P* < 0.05, Student’s *t*-test).

Surprisingly, we also noted that change of leaf morphology appeared in all the *35S::GhFT1* transgenic plants. To decipher the function of *GhFT1* further, the *35S::GhFT1* transgenic line 5 and line 6 were used to observe their leaves phenotype under LD and SD conditions. We compared leaves morphology from apical to basal position among the transgenic lines and the WT tobacco siblings. The leave area in the transgenic lines was significantly smaller than in the WT plants. Under LD conditions, the leaves in line 5 appeared to be much longer and narrower than that in the WT plants, but leaves in line 6 appeared to be much shorter and wider (**Figure 4A**). We next measured the leaf length to width (L/W) ratio in the transgenic lines and WT tobacco plants, respectively. Accordingly, line 5 had the largest L/W ratio value, followed by the WT plant and Line 6, respectively (**Figure 4B**). Strikingly, the leaves of all the *35S::GhFT1* transgenic lines looked much more green and fleshy than the WT plants. We then measured chlorophyll content, suggesting both line 5 and line 6 had higher total chlorophyll content than the WT plants (**Figure 4C**). Similar phenotype was also observed in the *35S::GhFT1* transgenic lines in SD conditions (Supplementary Figure S5A). Leaf mass per area (LMA) is a key trait in plant growth and an important indicator of plant strategies, which is most closely correlated with a relative growth rate, and has been used wildly in plant ecology, agronomy, and forestry (Poorter et al., 2009). The transgenic lines showed higher LMA values than the WT plants (**Figure 4D**), contributing to more fleshy leaves compared with the WT plants in LD conditions. Similar to LD conditions, all the *35S::GhFT1* transgenic plants (line 15 and line 16 were shown as an example) had higher L/W ratio, chlorophyll content and LMA values than the WT under SD conditions (Supplementary Figure S5).

**FIGURE 5:**
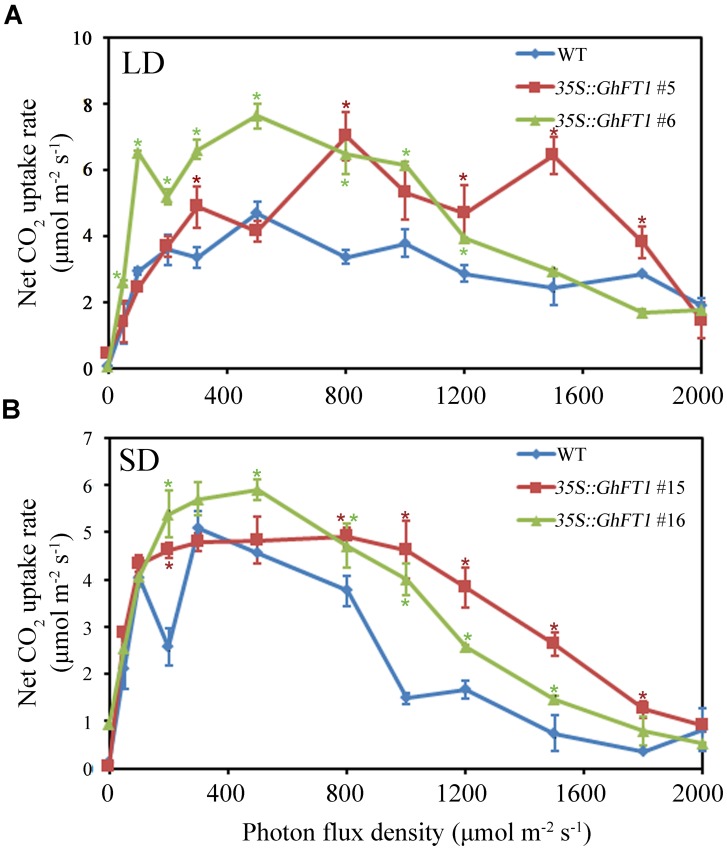
**Overexpression of cotton *GhFT1* increased photosynthetic efficiency in tobacco.** Photosynthetic efficiency among the WT and the *35S::GhFT1* transgenic tobacco lines was determined of using LI-6400 portable photosynthesis system at different light intensity under LD conditions **(A)** and SD conditions **(B)**, respectively. The WT plant and transgenic plants were 7 weeks-old after transfer to the phytotron. Data represent the mean ± SE of three independent experiments (*n* = 4). Asterisk denotes significant difference compared with WT plants at *P* < 0.05, according to the Student’s *t*-test, respectively.

We next set out to explore whether the change of leaf morphology and increasing of chlorophyll content in transgenic plants could enhance photosynthesis. The efficiency of photosynthesis was determined by LI-6400 portable photosynthesis system in different light intensity. As shown in **Figure 5**, the transgenic tobacco lines showed higher photosynthetic efficiency than the WT plant in LD as well as SD conditions. This suggests, *GhFT1* might play important roles in the modulation of leaf development in cotton by increasing photosynthesis and chlorophyll content other than floral transition.

### Ectopic Overexpression of *GhFT1* Caused Flowers Abscission

**FIGURE 6:**
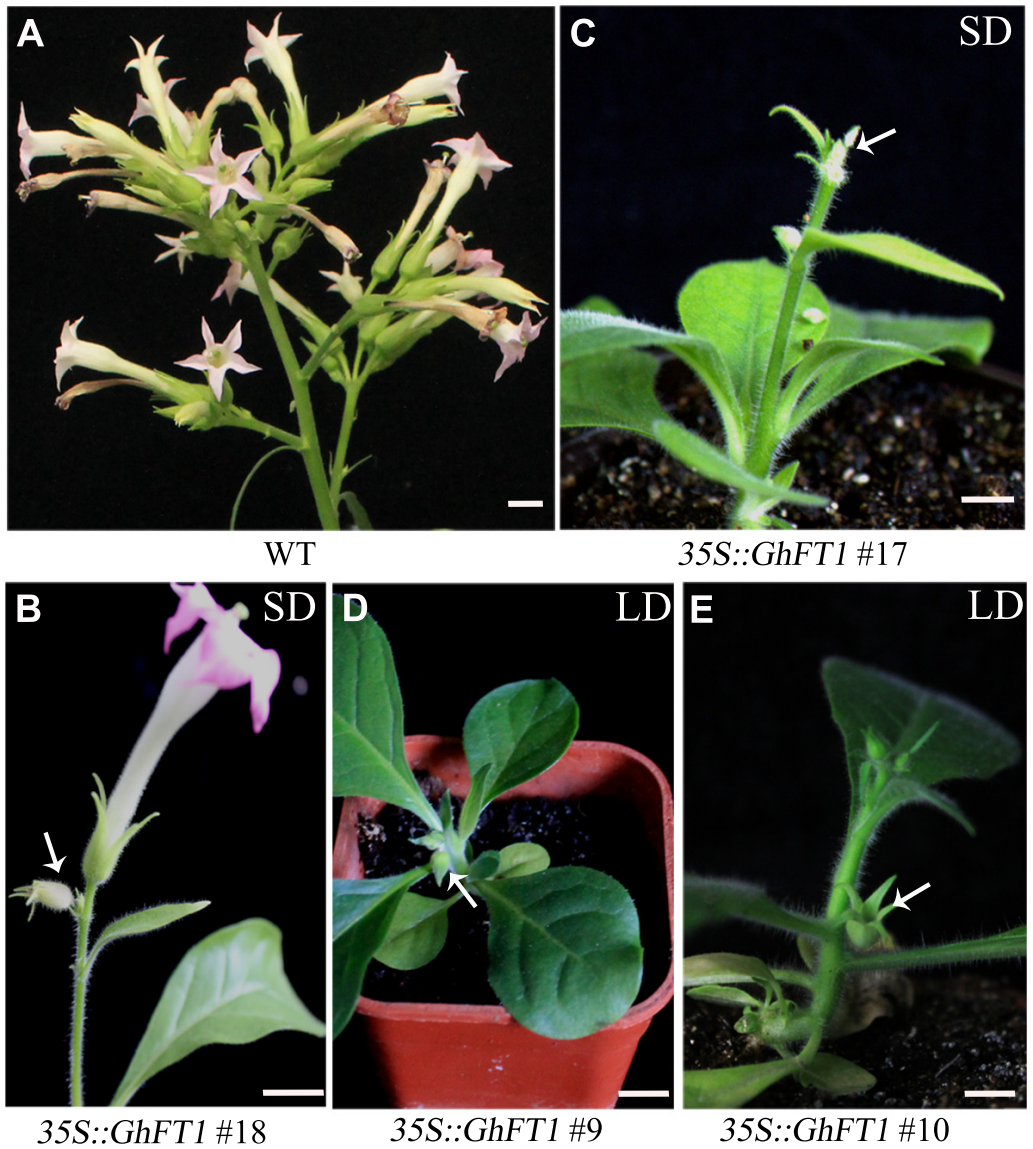
**Flower abscission behavior of transgenic tobacco lines that overexpressing *GhFT1* with severe phenotype. (A)** The cymose inflorescence in the WT flowers with no flowers abscission at flower opening. **(B)** The *35S::GhFT1* transgenic line 18 showed flowers abscission after the first flower opened under SD conditions. **(C)** The *35S::GhFT1* transgenic line 17 could not present the regular flowers, all of which abscised before opened under SD conditions. The *35S::GhFT1* transgenic tobacco line 9 **(D)** and line 10 **(E)** showed flowers abscission at the buds stage under LD conditions. Arrow indicated the abscised flower. Scale bars: 1 cm.

*Nicotiana tabacum* cv. NC89 is a typical cymose inflorescence in which the first-formed flower develops from the growing region at the top of the flower stalk, and the development of the flower at the apex is followed by two new flower axes developing from buds opposite on another (Amaya et al., 1999; **Figure 6A**). Both LD and SD conditions, strikingly, overexpression of *GhFT1* in tobacco caused extremely early flowering (**Figure 2**). In addition, 86% tobacco plants overexpressing *GhFT1* showed obvious premature flowering abscission in early flower developmental stages. For example, in the *35S:GhFT1* line 18, after the first flower opened, it abscised form the stalk (**Figure 6B**) under SD conditions, so it produced very few flowers and few mature seeds. In transgenic line 17, it could not present the regular flowers, due to their abscission before opening (**Figure 6C**). One of the reasons may be their flowers failed to enter meiosis, and eventually the plants did not produce any seed capsules. No cymose inflorescences similar to the WT plants (**Figure 6A**) were formed in all these transgenic lines. Similar to SD conditions, we also observed that these transgenic lines showed buds abscission at the initiation of early bud set under LD conditions (**Figures 6D,E**). However, the extent of floral bud abscission was alleviated, and flowers could normally open and seed set at later developmental stage (**Figures 2A,C**).

Viewed form outside, the transgenic plants showed normal flower development, produced fertile flowers and normal seeds (Supplementary Figures S6A,E). To investigate whether overexpression of *GhFT1* affected flower organs development in tobacco, we dissected the flowers of -1 days of anthesis (DOA) and 0 DOA in the WT and *35S::GhFT1* transgenic lines, respectively. For example, line 5 and line 6 showed a smaller flower than the WT control, but no difference of phenotype in stamen, stigma, petal, ovary, and sepal were observed (Supplementary Figures S6B–D), suggesting that the product of *GhFT1* had no influence on the development of flower organs.

### Influence of *GhFT1* Overexpression on the Expression Level of Other Genes in Tobacco

**FIGURE 7:**
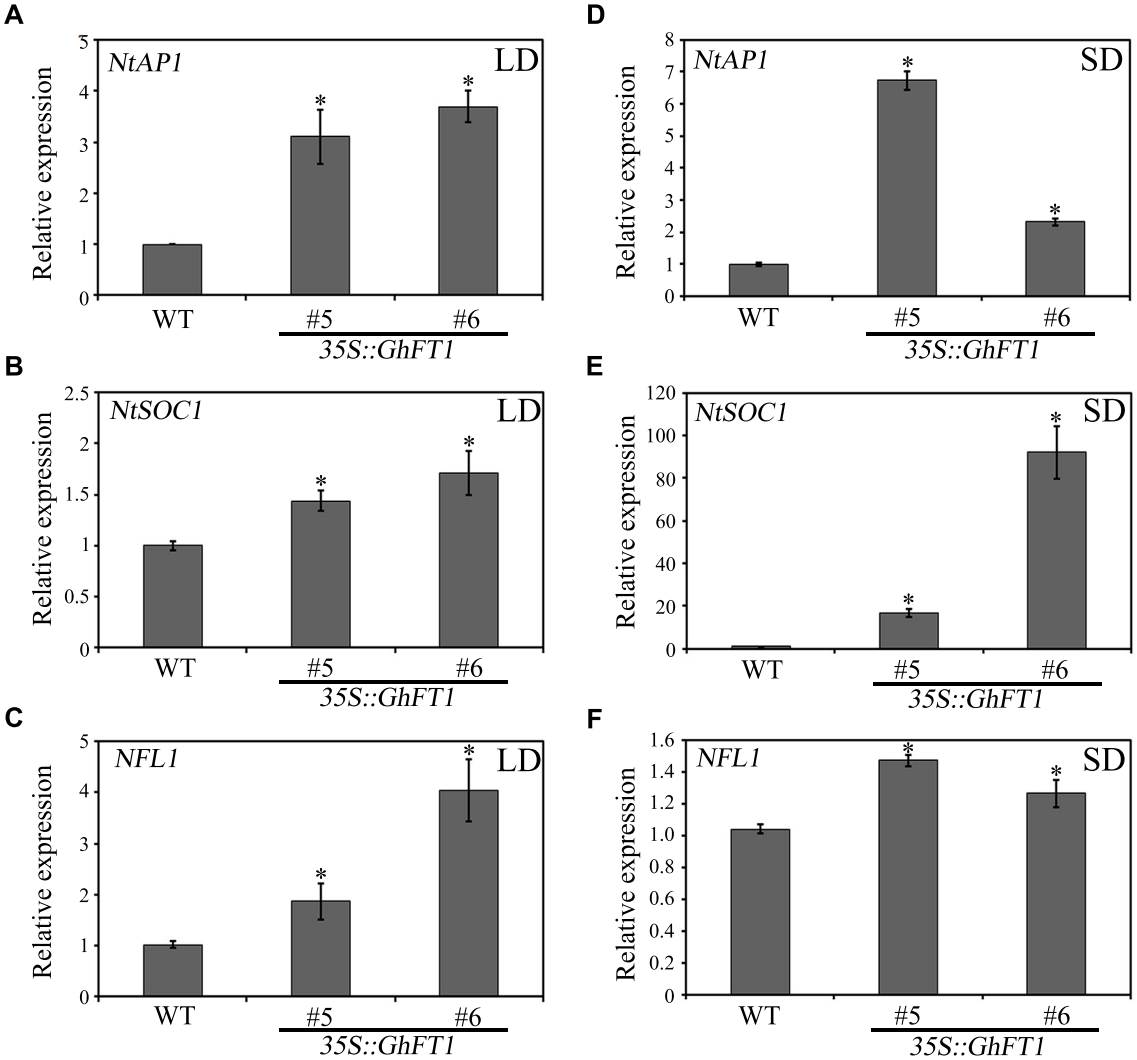
**Expression patterns of tobacco endogenous flowering-related gene in WT and *35S::GhFT1* transgenic lines.** Total RNA isolated from the *35S::GhFT1* transgenic tobacco line 5 and line 6 and one WT tobacco plant under LD and SD conditions was used as template, respectively. The expression level of *NtAP1* **(A,D)**, *NtSOC1* **(B,E)** and *NFL1* **(C,F)** was determined by qRT-PCR, respectively. Data represent the mean ± SE from three biological replicates (*n* = 3), and *NtActin* was used as internal control. *NtAP1*, JQ686939.1; *NFL1*, JQ686928.1; *NtSOC1*, JQ686938.1; *NtActin*, U60495. The asterisks indicate significant differences compared with the WT plants (*P* < 0.05, Student’s *t*-test).

In the present model, *FT* protein, is now widely established as a major component of florigen, a systemic signal that induces flowering in responsible to daylength, which is translocated through the phloem to the SAM (Corbesier et al., 2007; Mathieu et al., 2007; Notaguchi et al., 2008), where they form a complex involving a bZIP transcription factor FD to promote the transition to flowering by activating the expression of multiple flower meristem identity genes, such as *SOC1* and *AP1* (Abe et al., 2005; Fornara et al., 2010). The MADS-domain transcription factor *AP1* is a key regulator of *Arabidopsis* flower development, controlling the onset of flower development (Wigge et al., 2005; Kaufmann et al., 2010). *SOC1* integrates multiple flowering signals including photoperiod, temperature, hormone, and age-related signals, involving in the process of floral organ formation, meristem determinacy, and prevention of secondary growth and shoot longevity (Lee and Lee, 2010; Hiraoka et al., 2013). *LFY*, which encodes a plant specific transcription factor, plays dual roles in determining floral meristem identity and floral organ patterning via *AP1* and other floral homeotic genes (Moyroud et al., 2010). We next detected the expression profiles of the tobacco flower meristem identity genes among the transgenic and the WT siblings by qRT-PCR. The results indicated that *NtAP1*, *NFL* (the likely *Nicotiana FLO*/*LFY* homolog; Amaya et al., 1999) and *NtSOC1* were obviously upregulated in the transgenic lines under LD conditions (**Figures 7A–C**). Under SD conditions, the three genes were also obviously upregulated in *35S::GhFT1* transgenic tobacco plants (**Figures 7D–F**).

**FIGURE 8:**
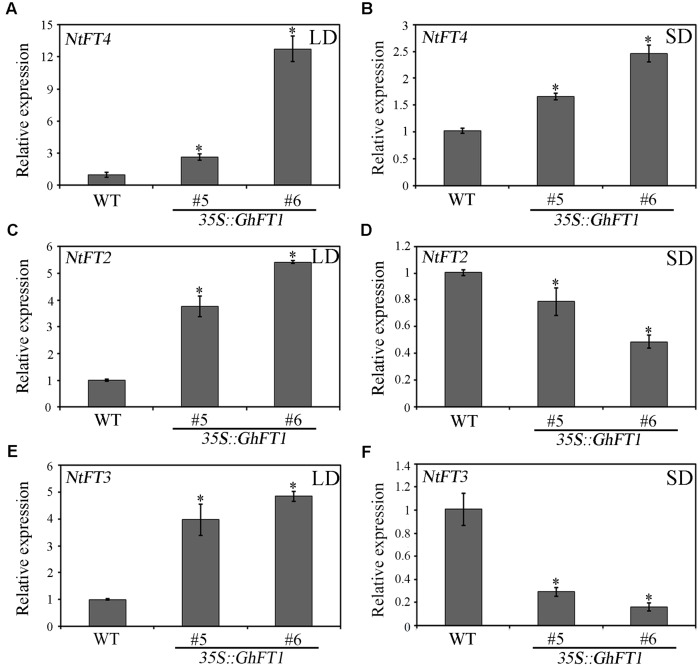
**Expression analysis for endogenous *FT* paralogs in tobacco plants that ectopically expressed *GhFT1*.** Total RNA isolated from the *35S::GhFT1* transgenic tobacco lines 5 and 6 and one WT tobacco plant under LD and SD conditions, was used as template to detect the expression of *NtFT4* **(A,B)**, *NtFT2* **(C,D)** and *NtFT3* **(E,F)** by qRT-PCR, respectively. Data represent the mean ± SE from three biological replicates (*n* = 3), with *NtActin* used as internal control. *NtFT2*, JX679068; *NtFT3*, JX679069; *NtFT4*, JX679070; *NtActin*, U60495. The asterisks indicate significant differences compared with the WT plants (*P* < 0.05, Student’s *t*-test).

Four *FT*-like genes have been identified in *N. tabacum* genome, *NtFT1*, *NtFT2*, *NtFT3* and *NtFT4*, which acts antagonistically to regulate floral initiation (Harig et al., 2012). The NtT1, NtFT2, and NtFT3 proteins are floral inhibitors, whereas NtFT4 is a floral inducer (Harig et al., 2012). To explore whether the early flowering phenotype was correlated with the endogenous *NtFTs* expression in *35S::GhFT1* transgenic lines, we next further detected the expressions profile of the four *FT* paralogs. As is shown in **Figure 8A**, higher *NtFT4* expression was observed in line 5 and line 6 than the WT plants under LD conditions. *NtFT4* was also observed highly expressed in transgenic tobacco plants under SD conditions (**Figure 8B**). Surprisingly, the expression of *NtFT2* and *NtFT3* were also upregulated in the *35S::GhFT1* transgenic line 5 and line 6 under LD condition (**Figures 8C,E**). However, both *NtFT2* and *NtFT3* were downregulated expressed in line 5 and line 6 in SD conditions (**Figures 8D,F**). It was previously reported that overexpression of *NtFT2* and *NtFT3* showed a delayed flower phenotype, but the exact biological functions of *NtFT2* and *NtFT3* remain unclear. We were unable to detect *NtFT1* expression under both conditions, while Harig et al. (2012) were unable to detect the expression of these genes under LD condition, which may be associated with the different tobacco varieties.

## Discussion

### Conserved Fuctions of FT-Like Proteins as Floral Promotes

A wide spectrum of research of *FT* orthologs from angiosperms has been demonstrated their conserved function in the regulation of flowering time (Pin and Nilsson, 2012). However, the developmental mechanisms targeted by *FT* orthologs to transform vegetative meristems into reproductive organs remain unclear. We previously identified a *FT*-like gene *GhFT1* from cotton (*G. hirsutum*), which was highly expressed in all the tissues except in root, and the strong sequence identity and critical amino acids residues Tyr88 (Y) and Gln144 (Q) to *FT*-orthologous genes of other species indicates that the *GhFT1* might be also involved in the control of flowering. Ectopic expression of *GhFT1* promoted precocious flowering under both LD and SD conditions in *Arabidopsis* (Guo et al., 2015), suggesting that GhFT1 is a potential *FT* ortholog that regulates floral transition in cotton. To investigate the developmental mechanism targeted by GhFT1 protein, we further unveiled its overall growth effects by overexpressing *GhFT1* in tobacco.

The transgenic tobacco plants carrying the *35S::GhFT1* construct flowered earlier and had fewer leaves at flowering than the WT plants in both LD and SD condition. Furthermore, the *5.7kbAtFTpro::GhFT1* construction by using 5.7-kb *Arabidopsis FT* gene promoter fused to the *GhFT1* cDNA could also accelerate flowering in transgenic tobacco (**Figure 2E**). The precocious flowering phenotype regardless of photoperiod indicated its conserved roles in floral induction.

Although *FT* orthologs have been identified and characterized from numerous plant species, the subcellular distributions of many of them have not been clearly studied. Here, we examined the distribution of functional GhFT1-GFP fusion protein expressed in leaf epidermal cells of *N. benthamiana* or in hypocotyl of tobacco by transform with *35S::GhFT1-GFP*. In both cases, GhFT1-GFP was observed in the nucleus and cytoplasm (**Figure 1**; Supplementary Figure S2), which was consistent with Guo et al. (2015) results. Similar results using a GFP or YFP-tagged *FT* were also reported in *Arabidopsis* (Abe et al., 2005), rice (Taoka et al., 2011), tomato (Lifschitz et al., 2006), and tobacco (Harig et al., 2012).

FT-GFP fusion protein in transgenic plant has been detected to move through the phloem from the leaves as the place of light perception to the shoot apex as the position of flower formation only in limited plant species (Supplementary Table S3). FT-GFP fusion proteins induced early flowering were previously reported in *Arabidopsis* (Corbesier et al., 2007), rice (Tamaki et al., 2007), and tomato (Shalit et al., 2009). However, the size of the fusion protein restricted the long-range function of FT. The larger FT-GFP protein may move less effectively to the SAM from the minor veins than from the larger veins (Supplementary Table S3). The *35S::GhFT1* with C-terminal translational fusion of GFP induced precocious flowering in tobacco, indicating GhFT1-GFP protein has similar activity like the WT *FT* protein. Previously publication have shown that overexpression of *FT* orthologs in tobacco could accelerate flowering in different plant species (Supplementary Table S4), including tomato (Shalit et al., 2009), fig (Ikegami et al., 2013), spring orchid (Xiang et al., 2012), London plane (Zhang et al., 2011), and tobacco itself (Harig et al., 2012; Guo et al., 2015; Wickland and Hanzawa, 2015).

Gene expression using qRT-PCR analysis revealed that the expression of *NAP1*, *NtSOC1*, and *NFL1* were significantly more upregulated in transgenic lines than in the WT plants under LD or SD conditions (**Figure 7**). These results were consistent with the finding that *NAP1* and *NFL1* were highly expressed in the flower buds of *35S::CgFT* tobacco plants (Xiang et al., 2012). These data indicates that the expression of *NAP1*, *NtSOC1*, and *NFL1* may be regulated by *FT*. Therefore, *GhFT1* might have upregulated them to regulate flowering in transgenic tobacco plants.

### Beyond Flowering Promotion: Pleiotropic Functions of GhFT1

Tobacco is a determinate species in which main shoot terminates by converting into a flower, with subsequent growth occurring only from lower meristems (**Figure 3A**). A number of axillary meristems generated below the apex also develop into terminal flowers in a cymose pattern (Amaya et al., 1999). Although florigen was originally proposed as a flowering hormone, it is now apparent that *FT* is a universal growth factor affecting several aspects of plant architecture. In addition to promoting flowering, we observed that the transgenic tobacco plants showed pleiotropic phenotype different from the WT control, suggesting that *GhFT1* played multifaceted roles during plant development.

That *Arabidopsis FT* is involved in the promotion of lateral shoot outgrowth and axillary bud initiation were previously proposed (Hiraoka et al., 2013; Niwa et al., 2013), but the single overexpression of *Arabidopsis FT* gene is insufficient to promote initiation or early development of axillary buds, and must combined with *LFY* (Niwa et al., 2013). However, ectopic overexpression of *GhFT1* in tobacco resulted in more axillary buds transition (**Figures 3B,D**) and more lateral shoots generation at the base of main shoot (**Figures 3C,E–G**; Supplementary Figure S4).

It has been previously reported that elevation of *FT* concentration promotes more determinate habit, and influences leaf development (Shalit et al., 2009; McGarry and Ayre, 2012). Here, we also observed that leaf morphology of the *35S::GhFT1* transgenic tobacco lines was very different from that of WT. Firstly, the leaves of transgenic lines appeared much more dark green and fleshy compared with WT plants. Accordingly, the chlorophyll content in transgenic lines was higher than that in the control plants (**Figure 4C**; Supplementary Figure S5C). Secondly, compared with other WT plants, leaves were shorter and wider in some transgenic lines, whereas leaves were longer and narrower in other transgenic lines. However, both had bigger value of L/W ratio and LMA under LD and SD conditions than the WT plants (**Figures 4B,D**; Supplementary Figures S5B,D). Likewise, transgenic lines showed higher photosynthetic efficiency than the WT plants (**Figure 5**). The results suggested that high *GhFT1* level could function to modulate leaf development by increasing L/W ratio, LMA and photosynthesis, and developing into smaller leaf. We surmised that *GhFT1* could link the transition to floral with leaf development.

The leaf morphological change in the *35S::GhFT1* transgenic tobacco lines is reminiscent of resent reports on overexpression *FT* orthologs in different plant species. It was previously reported that the florigen-dependent SFT/SP regulatory hierarchy could determine leaf architecture in tomato and overexpression of *SFT* induced simple lanceolate-like leaves (Shalit et al., 2009). When *Arabidopsis FT* is ectopically overexpressed in ancestral cotton accession TX701 through virus-induced flowering, it also generated the lanceolate leaf shape (McGarry and Ayre, 2012). Endo et al. (2005) reported that constitutive expression of *CiFT* in trifoliate orange altered the leaf shape and color; the leaf in the transgenic plants containing *35S:CiFT* was small, lacked color on the margin, and had a leaflet at the center of trifoliate leaf that was smaller than other leaflets. Xiang et al. (2012) reported that transgenic tobacco lines expressing *CgFT* showed the early release of axillary buds, and the rapid elongation of internodes enabled the formation of thinner stems and reduced leaf sizes. Teper-Bamnolker and Samach (2005) reported that overexpression of *Arabidopsis FT* induced the high level expression of *FUL* and *SEPTAL* (*SEP3*) in *Arabidopsis* and leaded to small-sized leaves. Overexpression of tobacco *NFL1* in tobacco results in dwarf stature, reduced internode length, and thickened leather-like leaves (Ahearn et al., 2001). Furthermore, Flachowsky et al. (2010) reported that ectopic expression of *Arabidopsis LFY* in apple showed an altered phenotype, which is similar to the columnar phenotype, and leads to shortened internodes and a significantly reduced length of the regrowing shoot. The high expression of *NFL1* (**Figure 7**) in tobacco driven by the overexpression of *GhFT1* might contribute to the leaf shape and plant architecture. Ectopically overexpressed transgenic plants containing *FT* orthologous genes exhibited similar phenotype in leaf shape and plant architecture, suggesting that the function of *FT*-like gene family is highly conserved during evolution.

Surprisingly, we also observed obvious premature flowering abscission in early developmental stages in the extremely early flowering transgenic lines carrying *35S::GhFT1*, resulting in fewer mature seeds (**Figure 6**). In tomato, *SFT* was also reported to accelerate mature and promote abscission zone formation (Shalit et al., 2009). Overexpression of *Arabidopsis FT* in the ancestral cotton accession TX701 delivered by virus-induced flowering caused many of flowers abscission before producing mature bolls (McGarry and Ayre, 2012). The abscission trait is considered as an innovation in angiosperms, and is regulated by multifactor, including auxin, ethylene, and jasmonic acid even day length (Shalit et al., 2009). Further study would clarify the possible mechanism for the precocious floral organ abscission in tobacco plants overexpressing cotton *GhFT1*.

### Overexpression of *GhFT1* Might Disturb the Balance between Inducer and Repressor of *FT* Parologs in Tobacco

It is now apparent that the relative ratios of *FT* to other members of the PEBP gene family have influenced the balance of indeterminate and determinate growth in many plant species, and play important role in the floral transition and architecture formation. For example, the tomato SFT/SP ratio regulates the reiterative growth and termination cycles typical of perennial plants, accelerates leaf maturation, influences the complexity of compound leaves, the growth of stems and the formation of abscission (Shalit et al., 2009). The recent report showed *N. tabacum* possesses four FT-like proteins (Harig et al., 2012), suggesting that the balance of *FT*-clade in tobacco plays important roles in the floral transition and plant architecture. However, the exact mechanism in the control of floral transition remains very unclear.

As expected, *NtFT4* showed significantly upregulated expression in all the transgenic tobacco plants under both light conditions (**Figures 8A,B**). Surprisingly, the expression of *NtFT2* and *NtFT3* were upregulated in transgenic plants under LD light conditions, but their expression levels were downregulated in SD conditions (**Figures 8C–F**). The contrast expression profiles of *NtFT2* and *NtFT3* under LD and SD conditions leads us to have a profound consideration of the florigen paradigm for influencing plant architecture. The balance model predicts that *FT* and TFL1 concentration fluctuate, and balance are re-fined in local tissues to give rise to different architecture (McGarry and Ayre, 2012). The expression levels of *GhFT1* in LD condition tobaccos were much higher than that in SD condition (**Figure 2**), suggesting that in LD and SD conditions, tobacco might have different concentration of florigen for mediating floral transition. High expression of cotton *GhFT1* completely influenced the expression level of endogenous *FT* genes in tobacco. As a floral inducer, *NtFT4* expression was highly upregulated under both LD and SD conditions, whereas *NtFT2* and *NtFT3* expression, acting as a floral repressor, were upregulated in LD condition and downregulated in SD, resulting in disorders of the balance between inducers and repressors in transgenic tobacco plants, therefore influences the *FT/TFL1* genes expression, and further changes FT/TFL1 proteins concentration in the transgenic tobacco. The problem balance will further influence the expression levels of flowering meristem identity genes, such as *NAP1*, *NFL1*, and *NtSOC1* (**Figure 7**), resulting in developing multifaceted phenotypes: early flowering, axillary buds set, lateral shoot outgrowth, leaf development change and flower abscission. However, further extensive research is needed to clarify these scenarios.

Taken together, overexpression of cotton *GhFT1* in tobacco promotes precocious flowering uncouple from photoperiod, showing that *FT* paralog evolves a conserved function of floral promoter in fiber plants. Introducing of transgenic cotton *FT* disturbs the balance of endogenous *FT* paralogs including inducers and repressors, and further disturbs other PEBP family members balance through antagonistic functions. We here present evidences that sufficient levels of *FT* activity might modulate axillary and lateral shoot outgrowth, influence leaf development and promote flower abscission, supporting the view that florigen functions as general growth hormone mediating growth and termination. These finding further extends the knowledge for plant florigen. Judicious manipulation of the ratio for indeterminate and determinate growth factors, mediated by a balance of *FT*-like and *TFL1*-like gene activities by transgenic technology, holds promise for improved plant architecture optimized for region-specific environment and enhanced crop yield in order to meet the agricultural demand of the rapidly expanding global population.

## Author Contributions

XH and XW designed the experiments and organized the manuscript. CL, YZ, KZ, and DG performed the experiments. XW and BC edited the manuscript. All the authors discussed the results and contributed to the manuscripts.

## Conflict of Interest Statement

The authors declare that the research was conducted in the absence of any commercial or financial relationships that could be construed as a potential conflict of interest.

## Supplementary Material

The Supplementary Material for this article can be found online at: http://journal.frontiersin.org/article/10.3389/fpls.2015.00454
