# Supplementary material for: Promoting flowering, lateral shoot outgrowth, leaf development, and flower abscission in tobacco plants overexpressing cotton FLOWERING LOCUS T (FT)-like gene GhFT1
Source: Front Plant Sci. 2015 Jun 17;6:454. doi: 10.3389/fpls.2015.00454 (PMC4469826; doi:10.3389/fpls.2015.00454)
Supplement: Supplementary file 1 [file Presentation_1.PDF]

Supplementary Material

Promoting Flowering, Lateral Shoot Outgrowth, Leaf Development, and Flower Abscission in Tobacco Plants Overexpressing Cotton *FLOWERING LOCUS T* (*FT*)-Like Gene *GhFT1*

Chao Li[1], Yannan Zhang[1], Kun Zhang[1], DanLi Guo[1], Baiming Cui[1], Xiyin Wang[2,*] and Xianzhong Huang[1,2*]

[1]Plant Genomics Laboratory, College of Life Sciences, Shihezi University, Shihezi, China
[2]Plant Genome Mapping Laboratory, University of Georgia, Athens, GA, USA

*** Correspondence:** Dr. Xianzhong Huang, [1]Plant Genomics Laboratory, College of Life Sciences, Shihezi University, North 4 RD 221, Shihezi, Xinjiang, 832003, China; [2]Plant Genome Mapping Laboratory, University of Georgia, Riverbend RD 111, Athens, GA, 30602, USA
e-mail: xianzhongh106@163.com
*** Correspondence:** Dr. Xiyin Wang, Plant Genome Mapping Laboratory, University of Georgia, Riverbend RD 111, Athens, GA, 30602, USA
e-mail: wang.xiyin@gmail.com

1. Supplementary Figures and Tables

Supplementary Tables 1-5

Supplementary Figures 1-6

Supplementary References

1.1. Supplementary Tables

Supplementary Table 1. The FT orthologs possessing floral inductive function from woody perennials, grasses, legumes and ornamentals plant species.

Classification	Gene	GenBank Accession No.	Origin	References
woody perennials	*CiFT*	AB027456	citrus	Hisada et al., 1997; Endo et al., 2005
	MdFT1/2	AB458506 AB458504	apple	Kotoda et al., 2010
	PdFT2	AY515152	Poplar deltoides	Hsu et al., 2006
	PtFT1	DQ387859	Poplar tremula	Böhlenius et al., 2006
	VcFT	CV091156	blueberry	Song et al., 2013
	VvFT	DQ504308	grapevine	Carmona et al., 2006
grasses	*BdFTL1/2*	KF572023 KF572024	*Brachypodium*	Wu et al., 2013
	Hd3a	AB052942	rice	Tamaki et al., 2007
	HvFT1/2/3	DQ100327 DQ297407 DQ411319	barley	Kikuchi et al., 2009
	ScFT1	KJ496327	sugarcane	Coelho et al., 2014
	TaFT	DQ890162	wheat	Yan et al., 2006
	ZCN8	EU241899	maize	Meng et al., 2011
legumes	*GmFT2a/5a*	KJ607992 KJ607997	soybean	Kong et al., 2010
	LjFT	GO011964	lotus	Ono et al., 2010
	MtFTa1	HQ721813	Medicago	Laurie et al., 2011
	PsFTa1	HQ538822	pea	Hecht et al., 2011
ornamentals	*AcFT2*	JX145039	onion	Lee et al., 2013
	BvFT2	HM448912	sugar beet	Pin et al., 2010
	CgFT	JX316221	spring orchid	Xiang et al., 2012
	CsFTL3	AB679272	chrysanthemum	Oda et al., 2012
	GtFT1	AB605176	gentian	Imamura et al., 2011
	LsFT	AB602322	lettuce	Fukuda et al., 2011
	NFT1	JX316221	Chinese narcissus	Li et al., 2013
	NtFT4	JX679070	tobacco	Harig et al., 2012
	OnFT	EU583502	Orchid	Hou and Yang, 2009
	PaFT	KM023154	avocado	Ziv et al., 2014
	PnFT	EU178859	Pharbitis	Hayama et al., 2007
	StSP3D	AY186735	tomato	Navarro et al., 2011

Supplementary Table 2. Flowering phenotype of transgenic tobacco lines expressing *GhFT1*.

Lines (Genotype)	Light conditions	Number of plants	Days of flowering[a]	Number of leaves[b]	Plant height (cm)[c]
WT	LD	30	106.3 ±4.8	16 ±0.8	20.55 ±1.92
35S::GhFT1	LD	14	45.8 ±4.8**	8 ±0.7**	6.49 ±1.8**
WT	SD	28	94.5 ±3.3	15.7 ±0.5	16.2 ±0.54
35S::GhFT1	SD	12	57.2 ±4.9**	7 ±0.9**	5.4 ±1.75**
35S::GhFT1-GFP	SD	12	63 ±6.6*	14.5 ±0.6	29.5 ±1.08*
5.7kbAtFTpro::GhFT1	SD	14	50.5 ±2.08*	13.5 ±1.0	27.4 ±1.66*

LD, long-day (16 h light/8 h dark); SD, short-day (8 h light/16 h dark). The data are presented as the mean ± SE from individual plants.

** and * indicate significant differences at $P < 0.01$ and $P < 0.05$ according to the Student's *t*-test compared with wild-type plants.

[a]days of flowering after being transplanted to soil;

[b]number of leaves before flowers;

[c]height of plants were measured when flower buds appeared.

Supplementary Table 3. Transgenic expression of *FT*-like genes using construct containing *FT:GFP* fusuion. SD, short-day (8 h light/16 h dark); LD, long-day (16 h light/8 h dark); *n*, number of leaves.

Gene	GenBank accession No.	Origin	Construct	Light conditions	acceptors	Flowering at *n* leaves (Days to flowering)	References
AtFT	At1G65480	*A. thaliana* (Col-0)	*SUC2:FT:GFP*	SD, LD	*ft-7*	4-7[a]	Kobayashi et al., 1999; Kardailsky et al., 1999 Corbesier et al., 2007
			KNAT1:FT:GFP	LD	*ft-7*	4-8 [a]	
			FT:FT:GFP	LD	*ft-7*	5-8 [a]	
			GAS1:FT:GFP	LD	*ft-7*	15-16 [a, c]	
Hd3a	AB062676	Japonica rice (Norin 8)	*Hd3a::Hd3a:GFP*	SD	Wild type	32.8 ±11.2 [b]	Tamaki et al., 2007
			RPP16::Hd3a:GFP	SD	Wild type	14.8 ±3.3 [b]	
			rolC::Hd3a:GFP	SD	Wild type	19.5 ±13.6 [b]	
SP3D	AY186735	Tomato	*35S:SFT-GFP*	SD	Wild type	3-4 [a]	Shalit et al., 2009
			35S:SFT-RFP	SD	Wild type	3-4 [a]	

[a] Leaf numbers at flowering

[b] Days to flowering

[c] *GAS1:FT:GFP* was introduced into *ft-7* mutant, and transgenic plants were as late flowering as *ft-7* mutants mutant (Corbesier et al. 2007).

Supplementary Table 4. Transgenic overexpression of *FT*-like genes in tobacco using construct containing *35S::FT*. SD, short-day (8 h light/16 h dark); LD, long-day (16 h light/8 h dark).

Origin	Gene	GenBank Accession No.	Promoter	Light conditions	acceptors	Phenotype	References
tomato (*Solanum lycopersicum*)	*SP3D*	AY186735	*35S*	SD, LD	Maryland Mammonth, Samsun	early flowering	Shalit et al., 2009
Fig (*Ficus carica*)	*FcFT1*	AB457620	*35S-Ω*	SD	Samsun NN	early flowering	Ikegami et al., 2013
spring orchid (*Cymbidium goeringii*)	*CgFT*	HM106985	*35S*	LD	*N. tabacum* cv. Yunyan 87	early flowering	Xiang et al., 2012
London plane (*Platanus acerifolia*)	*PaFT1A*	GQ847823	*35S*	LD	*N. tabacum* cv. Xathi (day-neutral)	early flowering	Zhang et al., 2011
tobacco (*N.tabacum* cv. SR1)	*NtFT1*	JX679067	*35S*	LD, SD	*N. tabacum* cv.SR1 (day-netural)	later flowering	Harig et al., 2012
	NtFT2	JX679068	*35S*		*N. tabacum* cv.SR1	later flowering	
	NtFT3	JX679069	*35S*	LD, SD	*N. tabacum* cv.SR1	later flowering	
	NtFT4	JX679070	*35S*	LD, SD	*N. tabacum* cv.SR1	early flowering	

Supplementary Table 5. Primers used in this study.

Primer	Sequence 5’ 3’	Used for
GhFT1-F2	TCTGCTATGAGAGCCCACGA	qRT-PCR for *GhFT1*
GhFT1-R2	TCATGTCCTACGGCCACCGGATCCACT	
NtAP1-F	TCAAAGAGGAGGGGAGGTTT	qRT-PCR for *NtAP1*
NtAP1-R	CAAGCGTCTCTCTGCGTATG	
NtSOC1-F	AGTTGGAGCGGAGTGTCAGT	qRT-PCR for *NtSOC1*
NtSOC1-R	GCCTTGTCTCTGTTGAAGGC	
NFL1-F	GGACAAGGGAGTTAGGAGGG	qRT-PCR for *NFL1*
NFL1-R	GAGAGCGTCAAGGGCATTAG	
NtFT1-F	AAGCAACCCAAACCTGAGGGAGTATCTG	qRT-PCR of *NtFT1*
NtFT1-R	GCAGCAACAGGCGAATTGAGATTATGAAATCTC	
NtFT2-F	AGATATCCCTGCAACCACAGAAGCAAC	qRT-PCR for *NtFT2*
NtFT2-R	AAACAGCGGCAACAGGCAAATTGAGAC	
NtFT3-F	AATTGTCCACCAACCTAGGGTTGACGTG	qRT-PCR for *NtFT3*
NtFT3-R	CATTCACAACATCTCGAGTCAATTGTCGAAACAG	
NtFT4-F	GATATCCCAGCAACTACAGATACAAG	qRT-PCR for *NtFT4*
NtFT4-R	GAAACGGGCAAACCAAGATTGTAAAC	
NtActin-F	AAGGGATGCGAGGATGGA	Internal control gene of qRT-PCR for *NtActin*
NtActin-R	CAAGGAAATCACCGCTTTGG	

1.2. Supplementary Figures

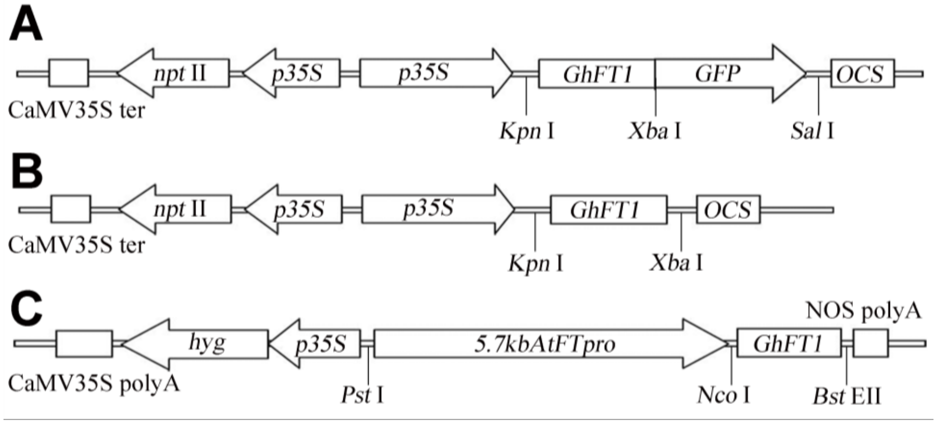

Supplementary Figure 1. Schematic representation of construct used for ectopically expressed *GhFT1* in tobacco with *35S::GhFT1* (A), *35S::GhFT1-GFP* (B) and *5.7kbAtFTpro::GhFT1* (C). CaMV35S: cauliflower mosaic virus 35S promoter; *npt* II: neomycin phosphotransferase II gene; *OCS*: octopine synthase gene; *NOS*: nopaline synthase gene.

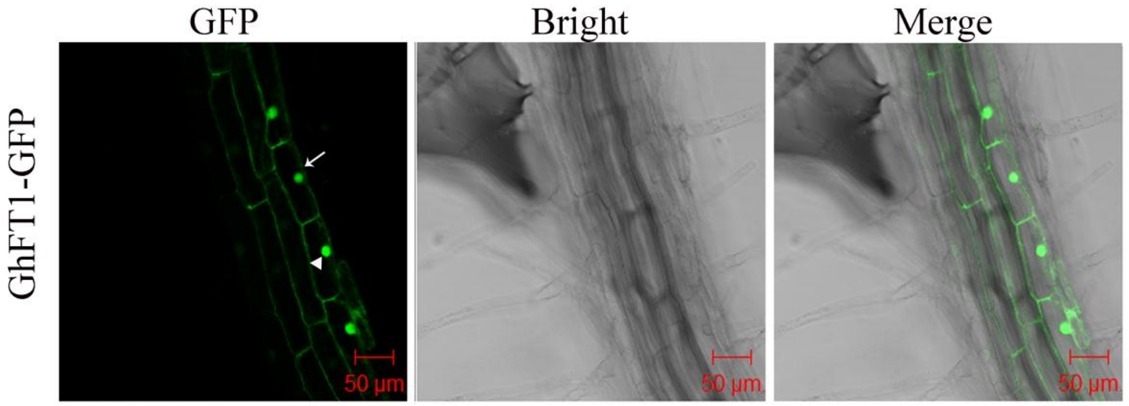

Supplementary Figure 2. Nucleus and cytoplasm subcelluar location of GhFT1-green fluorescent protein (GFP) in tobacco. Micrographs showing cells expressing GhFT1-GFP fusion protein in tobacco hypocotyl, which were examined under fluorescent-field illumination (left) to examine GFP fluorescence, and under bright-field illumination (middle), and by confocal microscopy (right) for an overlay of bright and fluorescent illumination. Arrow, plasma membrane; triangle, nucleus.

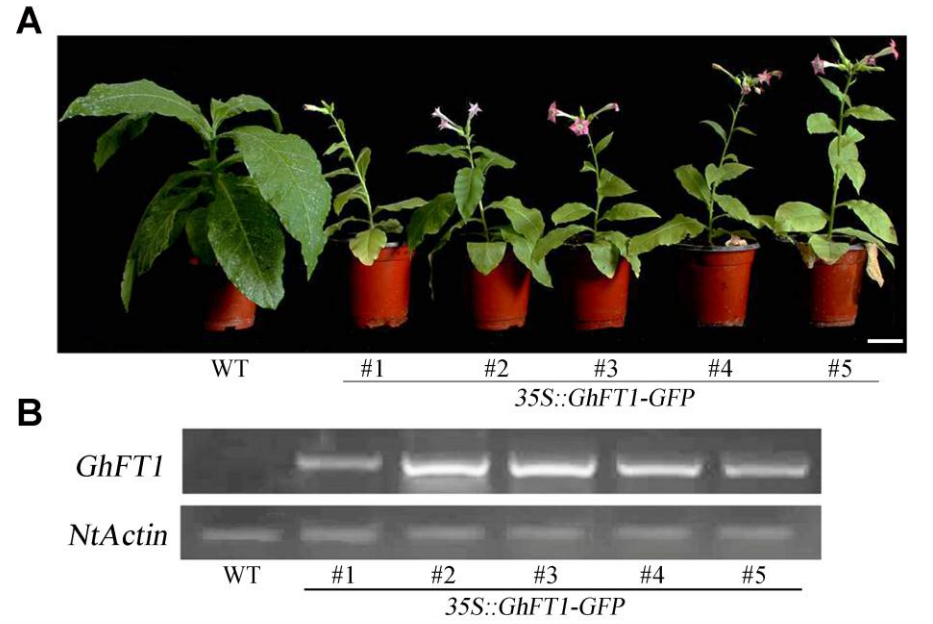

Supplementary Figure 3. Phenotype analysis of transgenic tobacco (*Nicotiana tabacum* cv. NC89) lines that ectopically overexpressed *GhFT1-GFP*. (A) Appearance of 52 d wild-type (WT) tobacco and *35S::GhFT1-GFP* transgenic tobacco lines grown in phytotron under short-day (SD) (8 h light/16 h dark) conditions. Scale bar: 7.5 cm. **(B)** Detection of *GhFT1* expression by semiquantitative reverse-transcription polymerase chain reaction (RT-PCR) in the *35S::GhFT1-GFP* transgenic lines and the WT control under SD conditions. Total RNA isolated from four *35S::GhFT1-GFP* transgenic tobacco lines and from one untransformed WT tobacco plant was used as template to detect the expression level of *GhFT1*.

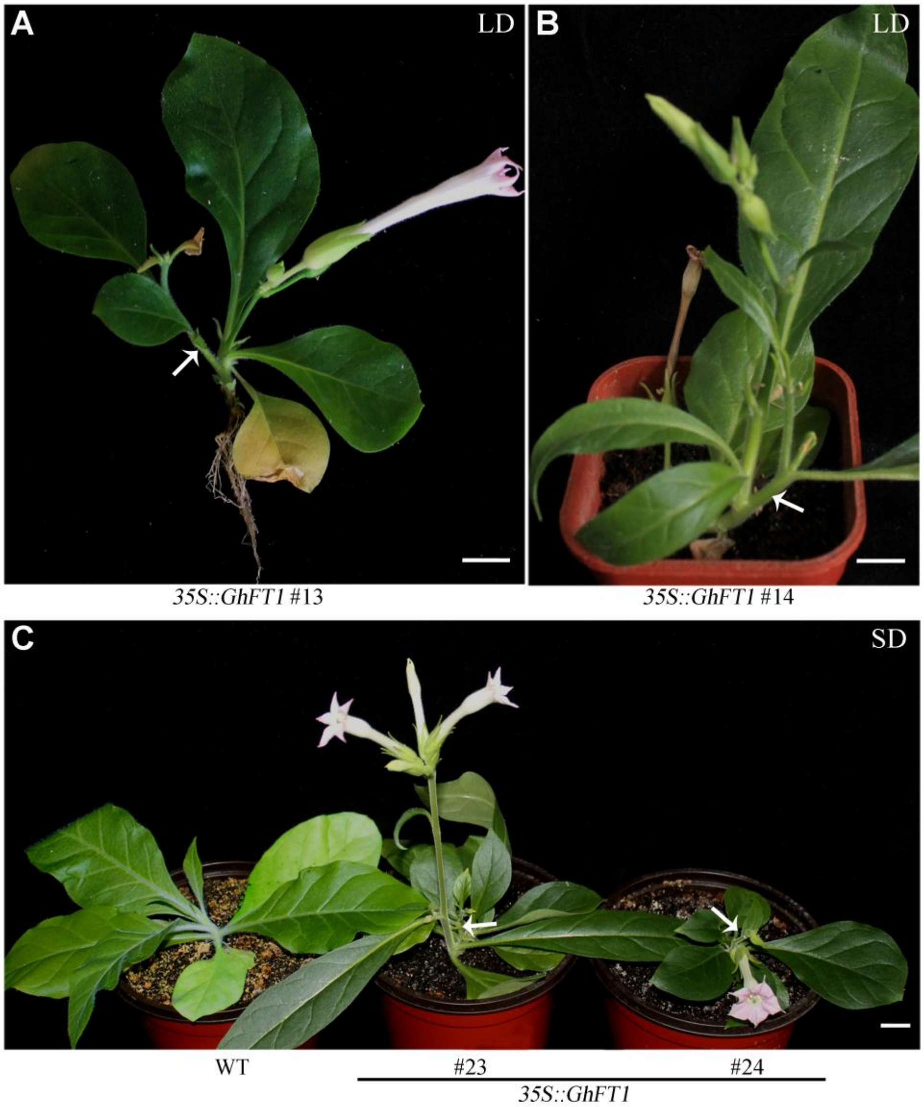

Supplementary Figure 4. Ectopic overexpression of *GhFT1* promotes lateral shoot outgrowth in growth. The homologous *35S::GhFT1* transgenic lines were planted in phytotron under long-day (LD) conditions (line 13) **(A)** and **(B)** (line 14), and short-day (SD) conditions (line 23 and line 24) (C), respectively. Arrows indicate the lateral shoot appeared from the base. Plants were photographed at 46 d after sowing in A and B, and 58 d after sowing in C. Scale bars: 1 cm.

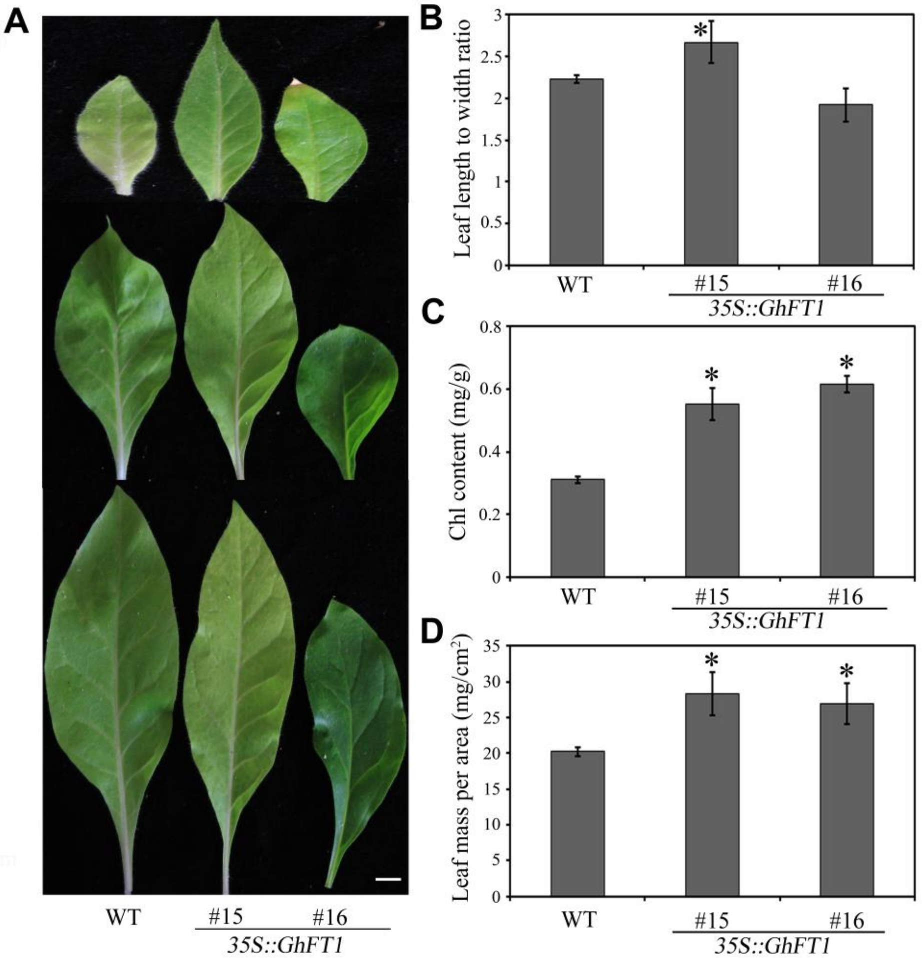

Supplementary Figure 5. Overexpression of *GhFT1* in tobacco exerted a strong influence on leaf morphology under short-day (SD) conditions. (A) Comparison of apical (top), medial (middle two), and basal (bottom) leaves between one wild-type plant and the *35S::GhFT1* transgenic line 15 and line 16 (at 7 weeks) under SD conditions. Scale bar, 1 cm. The ratio of leaf length to width (l/w) **(B)**, chlorophyll content **(C)** and Leaf mass per area (LMA) **(D)** were determined between a wild-type plant and the *35S::GhFT1* transgenic line 15 and line 16 (at 7 weeks) under SD conditions, respectively. Data represent the mean ± SE (n=4). Asterisks indicate significant differences between transgenic lines and WT plants ($P < 0.05$, Student's *t*-test).

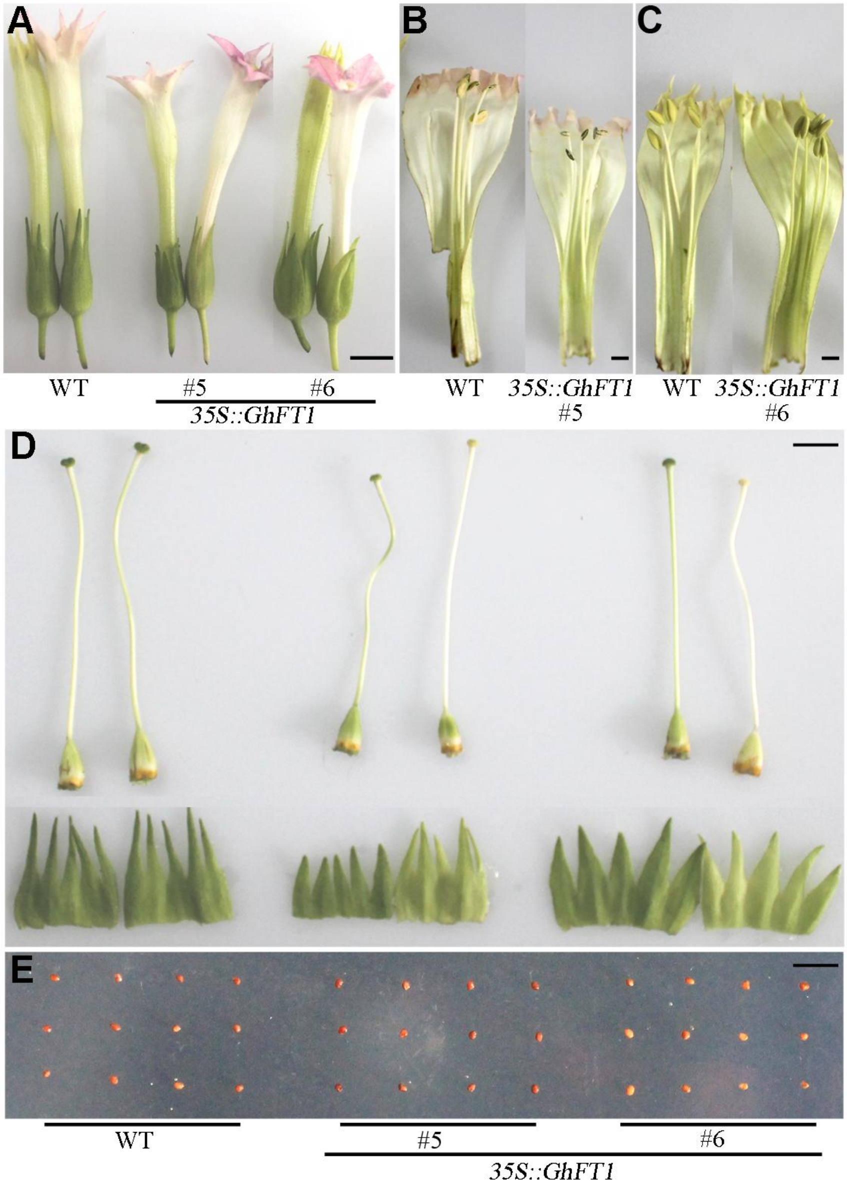

Supplementary Figure 6. Comparison of flower traits among the *35S::GhFT1* transgenic tobacco line 5 and line 6 and wild control. (A) Comparison of flowers appearance among the wild-type (WT) control and transgenic lines. -1 d of anthesis (DOA) (left) and 0 DOA (right). Comparison of stamens between WT and line 5 **(B)**, WT and line 6 **(C)**. **(D)** Comparison of ovary (top), and sepal (bottom). **(E)** Comparison of seed morphology. Scale bars: 1 cm.

SUPPLEMENTARY REFERENCES

Böhlenius, H., Huang, T., Charbonnel-Campaa, L., Brunner, A.M., Jansson, S., Strauss, S.H., et al. (2006). CO/FT regulatory module controls timing of flowering and seasonal growth cessation in trees. *Science* 312, 1040–1043. doi: 10.1126/science.1126038

Carmona, M.J., Calonje, M., and Martínez-Zapater, J.M. (2007). The FT/TFL1 gene family in grapevine. *Plant Mol. Biol.* 639, 637–650. doi: 10.1007/s11103-006-9113-z

Coelho, C.P., Minow, M.A., Chalfun-Júnior, A., and Colasanti, J. (2014). Putative sugarcane FT/TFL1 genes delay flowering time and alter reproductive architecture in Arabidopsis. *Front. Plant Sci.* 5, 221. doi: 10.3389/fpls.2014.00221

Corbesier, L., Vincent, C., Jang, S., Fornara, F., Fan, Q., Searle, I., et al. (2007). FT protein movement contributes to long-distance signaling in floral induction of Arabidopsis. *Science* 316, 1030–1033. doi: 10.1126/science.1141752

Endo, T., Shimada, T., Fujii, H., Kobayashi, Y., Araki, T., and Omura, M. (2005). Ectopic expression of an FT homolog from Citrus confers an early flowering phenotype on trifoliate orange (Poncirus trifoliata L. Raf.). *Transgenic Res*. 14, 703–712. doi: 10.1007/s11248-005-6632-3

Fukuda, M., Matsuo, S., Kikuchi, K., Kawazu, Y., Fujiyama, R., and Honda, I. (2011). Isolation and functional characterization of the FLOWERING LOCUS T homolog, the LsFT gene, in lettuce. *J. Plant Physiol*. 168, 1602–1607. doi: 10.1016/j.jplph.2011.02.004

Harig, L., Beinecke, F.A., Oltmanns, J., Muth, J., Müller, O., Rüping, B., et al. (2012). Proteins from the FLOWERING LOCUS T-like subclade of the PEBP family act antagonistically to regulate floral initiation in tobacco. *Plant J.* 72, 908–921. doi: 10.1111/j.1365-313X.2012.05125.x

Harig, L., Beinecke, F.A., Oltmanns, J., Muth, J., Müller, O., Rüping, B., et al. (2012). Proteins from the FLOWERING LOCUS T-like subclade of the PEBP family act antagonistically to regulate floral initiation in tobacco. *Plant J.* 72, 908–921. doi: 10.1111/j.1365-313X.2012.05125.x.

Hayama, R., Agashe, B., Luley, E., King, R., and Coupland, G. (2007). A circadian rhythm set by dusk determines the expression of FT homologs and the short-day photoperiodic flowering response in Pharbitis. *Plant Cell* 19, 2988–3000. doi: 10.1105/tpc.107.052480

Hecht, V., Laurie, R.E., Vander Schoor, J.K., Ridge, S., Knowles, C.L., Liew, L.C., et al. (2011). The pea GIGAS gene is a FLOWERING LOCUS T homolog necessary for graft-transmissible specification of flowering but not for responsiveness to photoperiod. *Plant Cell* 23, 147–161. doi: 10.1105/tpc.110.081042.

Hisada, S., Akihama, T., Endo, T., Moriguchi, T., and Omura, M. (1997). Expressed sequence tags of Citrus fruit during rapid cell development phase. *J. Am. Soc. Hortic. Sci*. 122, 808–812. doi: 10.1186/1471-2229-10-222

Hou, C.J., and Yang, C.H. (2009). Functional analysis of FT and TFL1 orthologs from orchid (Oncidium Gower Ramsey) that regulate the vegetative to reproductive transition. *Plant Cell Physiol*. 50, 1544–1557. doi: 10.1093/pcp/pcp099

Hsu, C., Liu, Y., Luthe, D.S., and Yuceer, C. (2006). Poplar FT2 shortens the juvenile phase and promotes seasonal flowering. *Plant Cell* 18, 1846–1861. doi: 10.1105/tpc.106.041038

Ikegami, H., Nogata, H., Inoue, Y., Himeno, S., Yakushiji, H., Hirata, C., et al. (2013). Expression of FcFT1, a FLOWERING LOCUS T-like gene, is regulated by light and associated with inflorescence differentiation in fig (Ficus carica L.). *BMC Plant Biol*. 13, 216. doi: 10.1186/1471-2229-13-216

Imamura, T., Nakatsuka, T., Higuchi, A., Nishihara, M., and Takahashi, H. (2011). The gentian orthologs of the FT/TFL1 gene family control floral initiation in Gentiana. *Plant Cell Physiol*. 52, 1031–1041. doi: 10.1093/pcp/pcr055

Kardailsky, I., Shukla, V.K., Ahn, J.H., Dagenais, N., Christensen, S.K., Nguyen, J.T., et al. (1999). Activation tagging of the floral inducer FT. *Science* 286, 1962–1965. doi:

10.1126/science.286.5446.1962

Kikuchi, R., Kawahigashi, H., Ando, T., Tonooka, T., and Handa, H. (2009). Molecular and functional characterization of PEBP genes in barley reveal the diversification of their roles in flowering. *Plant Physiol.* 149, 1341–1353. doi: 10.1104/pp.108.132134

Kobayashi, Y., Kaya, H., Goto, K., Iwabuchi, M., and Araki, T. (1999). A pair of related genes with antagonistic roles in mediating flowering signals. *Science* 286, 1960–1962. doi: 10.1126/science.286.5446.1960

Kong, F., Liu, B., Xia, Z., Sato, S., Kim, B.M., Watanabe, S., et al. (2010). Two coordinately regulated homologs of FLOWERING LOCUS T are involved in the control of photoperiodic flowering in soybean. *Plant Physiol*. 154, 1220–1231. doi: 10.1104/pp.110.160796

Kotoda, N., Hayashi, H., Suzuki, M., Igarashi, M., Hatsuyama, Y., Kidou, S., et al. (2010). Molecular characterization of FLOWERING LOCUS T-like genes of apple (Malus × domestica Borkh.). *Plant Cell Physiol.* 51, 561–575. doi: 10.1093/pcp/pcq021

Laurie, R.E., Diwadkar, P., Jaudal, M., Zhang, L., Hecht, V., Wen, J., et al. (2011). The Medicago FLOWERING LOCUS T homolog, MtFTa1, is a key regulator of flowering time. *Plant Physiol*. 156, 2207–2224. doi: 10.1104/pp.111.180182

Lee, R., Baldwin, S., Kenel, F., McCallum, J., and Macknight, R. (2013). FLOWERING LOCUS T genes control onion bulb formation and flowering. *Nat. Commun.* 4, 2884. doi: 10.1038/ncomms3884

Li, X.F., Jia, L.Y., Xu, J., Deng, X.J., Wang, Y., Zhang, W., et al. (2013). FT-like NFT1 gene may play a role in flower transition induced by heat accumulation in Narcissus tazetta var. chinensis. *Plant Cell Physiol.* 54, 270–281. doi: 10.1093/pcp/pcs181

Meng, X., Muszynski, M.G., and Danilevskaya, O.N. (2011). The FT-like ZCN8 gene functions as a floral activator and is involved in photoperiod sensitivity in maize. *Plant Cell* 23, 942–960. doi: 10.1105/tpc.110.081406

Navarro, C., Abelenda, J.A., Cruz-Oró, E., Cuéllar, C.A., Tamaki, S., Silva, J., et al. (2011). Control of flowering and storage organ formation in potato by FLOWERING LOCUS T. *Nature* 478, 119–122. doi: 10.1038/nature10431

Oda, A., Narumi, T., Li, T., Kando, T., Higuchi, Y., Sumitomo, K., et al. (2012). CsFTL3, a chrysanthemum FLOWERING LOCUS T-like gene, is a key regulator of photoperiodic flowering in chrysanthemums. *J. Exp. Bot*. 63, 1461–1477. doi: 10.1093/jxb/err387

Ono, N., Ishida, K., Yamashino, T., Nakanishi, H., Sato, S., Tabata, S., et al. (2010). Genomewide characterization of the light-responsive and clock-controlled output pathways in Lotus japonicus with special emphasis of its uniqueness. *Plant Cell Physiol.* 51, 1800–1814. doi: 10.1093/pcp/pcq140

Pin, P.A., Benlloch, R., Bonnet, D., Wremerth-Weich, E., Kraft, T., Gielen, J.J., et al. (2010). An antagonistic pair of FT homologs mediates the control of flowering time in sugar beet. *Science* 330, 1397–1400. doi: 10.1126/science.1197004

Shalit, A., Rozman, A., Goldshmidt, A., Alvarez, J.P., Bowman, J.L., Eshed, Y., et al. (2009). The flowering hormone florigen functions as a general systemic regulator of growth and termination. *Pro. Nat. Acad. Sci. U.S.A.* 106, 8392–8397. doi: 10.1073/pnas.0810810106

Song, G.Q., Walworth, A., Zhao, D., Jiang, N., and Hancock, J.F. (2013). The Vaccinium corymbosum FLOWERING LOCUS T-like gene (VcFT): a flowering activator reverses photoperiodic and chilling requirements in blueberry. *Plant Cell Rep*. 32, 1759–1769. doi: 10.1007/s00299-013-1489-z

Tamaki, S., Matsuo, S., Wong, H.L., Yokoi, S., and Shimamoto, K. (2007). Hd3a protein is a mobile flowering signal in rice. *Science* 316, 1033–1036. doi: 10.1126/science.1141753

Tamaki, S., Matsuo, S., Wong, H.L., Yokoi, S., and Shimamoto, K. (2007). Hd3a protein is a mobile flowering signal in rice. *Science* 316, 1033–1036. doi: 10.1126/science.1141753

Wu, L., Liu, D., Wu, J., Zhang, R., Qin, Z., Liu, D., et al. (2013). Regulation of FLOWERING LOCUS T by a microRNA in Brachypodium distachyon. *Plant Cell* 25, 4363–4377. doi: 10.1105/tpc.113.118620

Xiang, L., Li, X., Qin, D., Guo, F., Wu, C., Miao, L., et al (2012). Functional analysis of FLOWERING LOCUS T orthologs from spring orchid (Cymbidium goeringii Rchb. f.) that regulates the vegetative to reproductive transition. *Plant Physiol. Biochem.* 58, 98–105. doi: 10.1016/j.plaphy.2012.06.011

Xiang, L., Li, X., Qin, D., Guo, F., Wu, C., Miao, L., et al. (2012). Functional analysis of FLOWERING LOCUS T orthologs from spring orchid (Cymbidium goeringii Rchb. f.) that regulates the vegetative to reproductive transition. *Plant Physiol. Biochem.* 58, 98–105. doi: 10.1016/j.plaphy.2012.06.011

Yan, L., Fu, D., Li, C., Blechl, A., Tranquilli, G., Bonafede, M., et al. (2006). The wheat and barley vernalization gene VRN3 is an orthologue of FT. *Proc. Natl. Acad. Sci. U.S.A.* 103, 19581–19586. doi: 10.1073/pnas.0607142103

Zhang, J., Liu, G., Guo, C., He, Y., Li, Z., Ning, G., et al. (2011). The FLOWERING LOCUS T orthologous gene of Platanus acerifolia is expressed as alternatively spliced forms with distinct spatial and temporal patterns. *Plant Biol*. (Stuttg). 13, 809–820. doi: 10.1111/j.1438-8677.2010.00432.x

Ziv, D., Zviran, T., Zezak, O., Samach, A., and Irihimovitch, V. (2014). Expression profiling of FLOWERING LOCUS T-like gene in alternate bearing 'Hass' avocado trees suggests a role for PaFT in avocado flower induction. *PLoS One* 9, e110613. doi: 10.1371/journal.pone.0110613
